# Repeated treatment with short-term mild stress reverses aging- and stress-induced emotional and social behavioral deficits

**DOI:** 10.1038/s12276-026-01641-2

**Published:** 2026-02-12

**Authors:** Eun-Hwa Lee, Jin-Young Park, Hyejin Kwon, So-Young Park, Pyung-Lim Han

**Affiliations:** 1 Ewha Medical Research Institute, College of Medicine, Ewha Womans University, Seoul, Republic of Korea; 2 Department of Brain and Cognitive Sciences, Scranton College, Ewha Womans University, Seoul, Republic of Korea

**Keywords:** Depression, Stress and resilience

## Abstract

Aging increases vulnerability to stress-induced neuronal dysfunction, yet the underlying mechanisms remain unclear. Here, in young mice (2 months), chronic stress elevates basal serum glucocorticoid (GC) levels and induces despair-like behavior, as well as impaired sociability. By contrast, aged mice (14.5 months) naturally exhibit elevated basal GC levels, but do not display depressive-like behavior or sociability deficits, although further analysis reveals a social memory impairment. However, exposure to subthreshold stress in aged mice further elevates basal GC levels and induces both emotional and sociability impairments. Notably, repeated mild stress reverses these stress-induced physiological and behavioral impairments in young and aged mice. Neural activity-dependent c-Fos expression mapping identifies the ventral subiculum (vSub) as a potential upstream neural hub that regulates both serum GC responses and emotional and social behaviors. Chemogenetic activation of the vSub, particularly the vSub-to-dorsal bed nucleus of the stria terminalis circuitry, reverses stress-induced increases in basal GC levels and the associated behavioral deficits. Transcriptomic analysis reveals that the vSub gene expression profile in aged mice significantly overlaps with that of young mice exposed to chronic stress, notably characterized by Fkbp5 upregulation. Targeted knockdown of *Fkbp5* within the vSub mitigates stress-induced increases in GC levels and rescues behavioral deficits. Moreover, repeated short-term mild stress or low-dose GC treatment ameliorates stress-induced physiological and behavioral impairments, accompanied by downregulation of *Fkbp5* in the vSub. Collectively, these results suggest that the aged brain acquires chronic stress-like signatures, heightening its vulnerability to maladaptive outcomes, and that repeated short-term mild stress can restore emotional and social function by normalizing vSub *Fkbp5*-dependent signaling.

## Aging brain shows stress vulnerability and recovery pathways

As we age, our brains undergo changes that make us more vulnerable to stress. This study explores how enhanced sensitivity to stress in aging brains can be overcome. Researchers have demonstrated that aging is accompanied by the accumulation of stress hormone-dependent changes in the brain, which can be reversed by a method known as ‘repeated mild stress’, involving short daily sessions of gentle restraint or rocking. Researchers measured changes in behavior and brain chemistry, focusing on specific brain areas such as the ventral subiculum, which are functionally downregulated in aged brains as in young mice under chronic stress. The study found that repeated mild stress helped reverse stress-induced behavioral problems and the reduced neural activity changes in both young and aged mice. This suggests that mild stress could be a potential therapy for stress-related issues in aging brains.

This summary was initially drafted using artificial intelligence, then revised and fact-checked by the author.

## Introduction

Brain aging involves various molecular and cellular alterations, including oxidative stress^1,2^, epigenetic dysregulation of gene expression^3,4^, and elevated levels of inflammatory factors^5,6^. These age-associated alterations increase vulnerability to stress^7–11^. However, the detailed mechanisms through which these processes arise and the manner in which aging and stress converge to influence brain function remain poorly understood.

Normal physiological stress activates the hypothalamus–pituitary–adrenal (HPA) axis, leading to the release of glucocorticoids (GCs)^12,13^. Released GCs stimulate cellular metabolism, facilitate essential bodily functions, and improve cognitive and behavioral responses^14,15^, thus being beneficial for daily life. However, chronic or excessive stress causes molecular and neuronal alterations over a homeostatic range, which results in neuronal dysfunction, and manifesting as impairments in cognitive function and emotional behaviors^14–16^. Stress-induced maladaptive changes within limbic structures, including the medial prefrontal cortex, hippocampus and amygdala, can impair emotional behaviors. Furthermore, these changes also affect the HPA axis, leading to elevated basal GC levels and altered HPA axis responses to acute stress challenges^7,12,17,18^. Intriguingly, repeated treatment with daily short-term restraint can reverse stress-induced elevated basal GC levels, and depressive-like behaviors in mice subjected to chronic stress^18^. These results prompt the question of whether such nonpharmacological, mild stress therapy could modify maladaptive changes associated with chronic stress in the aging brain.

The ventral subiculum (vSub), a key hippocampal output structure, projects its axons to the limbic structures, including the prefrontal cortex, nucleus accumbens and basolateral amygdala (BLA)^19,20^, facilitating the activity of the neural systems supporting emotional and cognitive processes^17,21^. The vSub also sends collaterals to the bed nucleus of the stria terminalis (BNST), which regulates the hypothalamic paraventricular nucleus (PVN)^16,20,22^. Thus, it is well established that the neuroanatomical organization of the vSub supports its role in regulating both HPA axis activity and emotional and cognitive behaviors. However, the molecular mechanisms underlying these functions remain poorly understood, and whether the vSub contributes to stress regulation in the aging brain has yet to be explored.

In the present study, we investigate the neural and molecular mechanisms of how aging increases stress-induced detrimental changes, and whether repeated mild stress could reverse chronic stress-induced maladaptive changes and their behavioral consequences in aged animals.

## Materials and methods

The details of the ‘Materials and methods’ are provided in the Supplementary Information.

### Animals

Male 7-week-old C57BL/6 mice were obtained from Daehan BioLink. Aged mice were maintained by housing two to four animals of the same sex per cage. All animal procedures were performed in accordance with Animal Ethics Committee of Ewha Womans University and were approved by the Institutional Animal Care and Use Committee at Ewha Womans University (IACUC19-015).

### Chronic and subchronic restraint stress

Subchronic or chronic restraint was performed as described previously^7,18^.

### Repeated 5-min restraint or 5-min gentle rocking stress

Repeated 5-min restraint was delivered as described previously^18^. The repeated 5-min gentle cage-shaking procedure was developed in the present study.

### Behavioral assay

Behavioral tests were carried out as described previously^18,23,24^. Throughout all behavioral testing, 65-dB white noise was used to mask background noise. Behavioral tests were recorded using either a video tracking system (SMART; Panlab S.I., Harvard Apparatus) or a webcam recording system (HD Webcam #C210; Logitech).

### RNA-seq analysis

RNA sequencing (RNA-seq) analysis was performed as described previously^18,25^. A total of 45,777 genes were included in the differential expression analysis.

Differentially expressed genes (DEGs) by aging or chronic restraint stress (CRST) were selected on the basis of a fold change threshold of ≥1.15 (upregulation or downregulation) and a significance level of *P* < 0.05. The differential gene expression for pairwise was determined using a rank–rank hypergeometric overlap (RRHO) method (http://systems.crump.ucla.edu/rankrank/)^26,27^.

### Gene knockdown and gene overexpression experiments in HT22 cells

Mouse HT22 cells were cultured as described previously^18^. Transfection of small interfering RNA (siRNA)-target gene and plasmid-Mecp2 DNA into HT22 cells was performed as described previously^7,28^.

### ChIP–quantitative PCR analyses

The chromatin immunoprecipitation (ChIP)–quantitative PCR assay was performed as described previously^7^.

### Serum corticosterone measurement

Serum corticosterone levels were quantified as described previously^18^ using an enzyme-linked immunosorbent assay kit.

### Chemogenetic manipulation of specific neuronal circuits

Chemogenetic modulation of neurons was carried out using a DREADD (designer receptors exclusively activated by designer drugs) system as described previously^18,27^.

### siRNA-mediated gene knockdown in the vSub

Stereotaxic injection of siRNA was performed as described previously^7,18^. siRNA-Fkbp5 or siRNA-control was injected into the vSub (anterior–posterior, −3.5 mm; medial–lateral, ±2.75 mm; dorsal–ventral, −4.5 mm) using a stereotaxic injection system (Vernier Stereotaxic Instrument).

### Statistical analysis

Two-sample comparisons were performed using two-sided Student’s *t*-test, and multiple comparisons were analyzed with one-way analysis of variance (ANOVA) followed by the Newman–Keuls post-hoc test. All data are presented as mean ± s.e.m., and statistical differences were accepted at *P* < 0.05 unless otherwise indicated. The statistical details of the results of all main figures and supplementary figures are provided in Supplementary Data 4 (details of statistical analysis).

## Results

### Repeated short-term mild stress reversed stress-induced emotional and social behavior deficits in aged mice

**Fig. 1:**
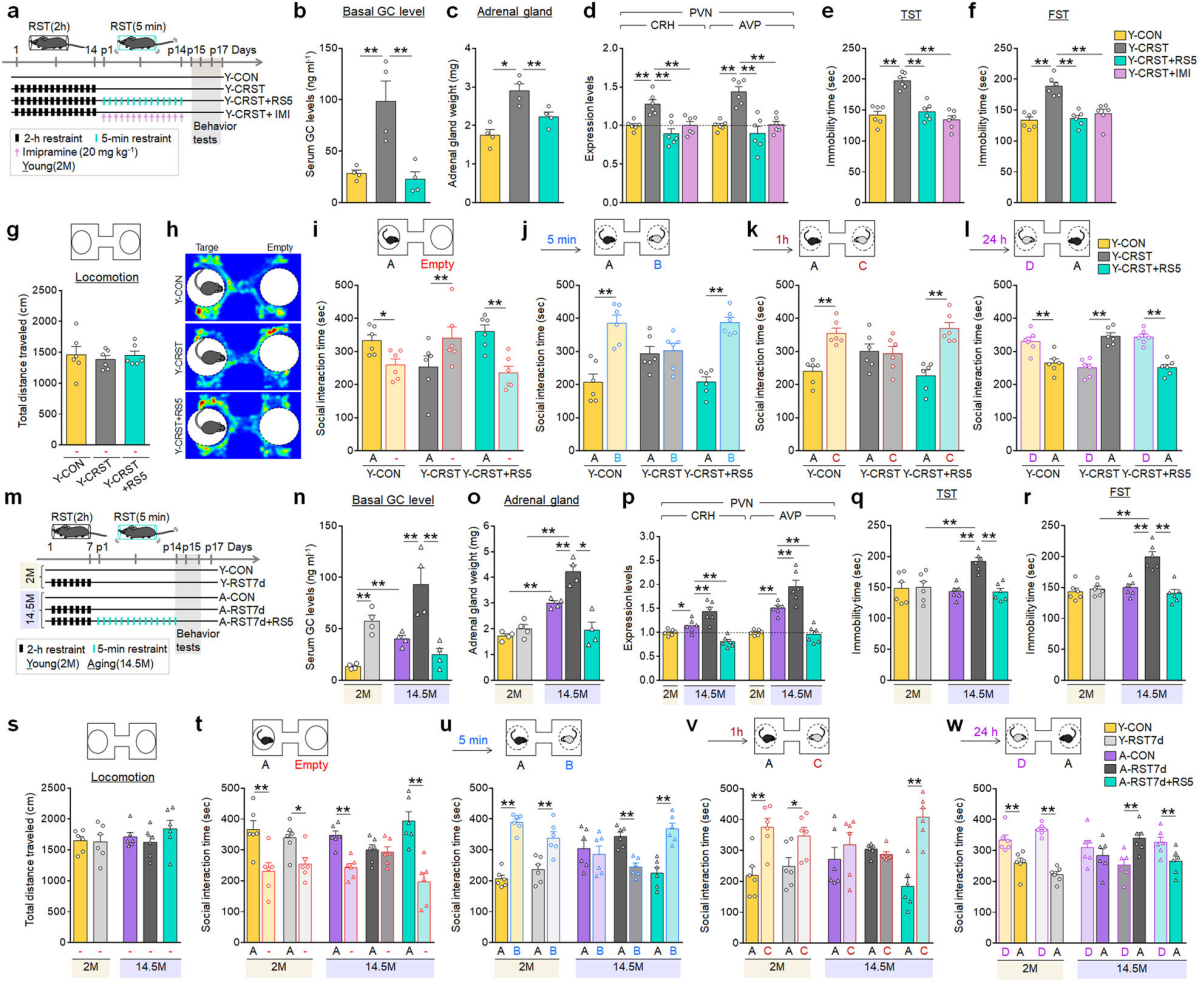
Repeated short-term mild stress reversed stress-induced depressive and social behavioral impairments. **a** Experimental design. Young mice (2 months, 2 M) subjected to CRST were treated with repeated 5-min restraint (RS5) or imipramine (IMI, 20 mg/kg, intraperitoneally (i.p.)) for 14 days (*n* = 6 animals per group). **b**–**d** Basal serum GC levels (**b**), adrenal gland weights (**c**) and CRH and AVP levels in the PVN (**d**) for the indicated groups. **e**–**l** Immobility time (s) in the TST (**e**) and FST (**f**). Social behavior tests: locomotor activity during habituation (**g**); heatmaps showing exploring activity (**h**); interaction time (**i**) with a target versus an empty in the SIT; and interaction time with familiar versus novel targets in the SMT at 5 min (**j**), 1 h (**k**) and 24 h (**l**) post-SIT for the indicated groups. **m** Experimental design. Young (2 M) and aged (14.5 months, 14.5 M) mice were subjected to 7-day 2-h restraint (RST7d) and subsequently with repeated 5-min restraint (RS5) for 14 days (*n* = 6 animals per group). **n**–**p** Basal serum GC levels (**n**), adrenal gland weights (**o**) and CRH and AVP levels in the PVN (**p**) for the indicated groups. **q**–**w** The immobility time in the TST (**q**) and FST (**r**). Sociability tests: the locomotion during habituation (**s**); interaction time (**t**) with a target versus an empty in the SIT; and interaction time with familiar versus novel targets in the SMT at 5 min (**u**), 1 h (**v**) and 24 h (**w**) post-SIT for the indicated groups. Data are mean ± s.e.m. *,**, difference between indicated groups. *P < 0.05; **P < 0.01 (Student’s *t*-test; one-way ANOVA followed by Newman–Keuls post-hoc test). See Supplementary Data 4 for statistical details.

Mice (2 months) subjected to 14 days of CRST, involving daily 2-h restraint, exhibited typical chronic stress signatures, including elevated basal serum GC levels, increased corticotropin-releasing hormone (CRH) and arginine vasopressin (AVP) expression in the PVN (Fig. 1a–f) and behavioral deficits such as despair-like behavior, impaired sociability and social memory impairments (Fig. 1g–l). However, repeated 5-min daily restraint for 14 days effectively reversed physiological and depressive-like behavior induced by CRST, a result similar to imipramine treatment (Fig. 1d–f) It also rescued CRST-induced social behavioral deficits (Fig. 1g–l).

Naive aged mice (14.5 months) presented with higher basal GC levels compared with young mice, but showed normal immobility in the tail suspension test (TST) and forced swim test (FST), and typical sociability. However, they displayed impaired social memory across all tested time intervals (5 min, 1 h and 24 h). Subchronic stress (RST7d; 2-h daily restraint for 7 days) did not induce despair-like behavior or social behavioral deficits in young mice. By contrast, aged mice subjected to RST7d exhibited increased basal serum GC levels, adrenal gland weights, and CRH and AVP expression in the PVN compared with young mice. RST7d also induced emotional and social behavioral deficits in aged mice (Fig. 1m–w), which were comparable to those observed in young mice subjected to CRST. However, repeated daily 5-min restraint for 14 days reversed RST7d-induced emotional and social behavioral deficits in aged mice (Fig. 1m–w). These results underscore the therapeutic potential of repeated mild stress for restoring aging- and stress-induced changes.

### Repeated mild stress effects required activation of the vSub

To explore the neural mechanisms activated by repeated mild stress, we mapped brain-wide c-Fos expression following repeated 5-min restraint sessions. This investigation identified several brain regions exhibiting similar activation patterns in both young (2 months) and aged (14.5 months) mice, including the vSub, ventral and dorsal hippocampal subregions, prelimbic cortex (PL), infralimbic cortex, dorsal BNST (dBNST), amygdala subregions and PVN (Supplementary Fig. 1). Despite the potential importance of other brain regions, we focused on the vSub because of its critical role in regulating stress coping and emotional behaviors, as well as its function as an upstream regulator of the PVN^16,22^.

To determine its potential role in regulating chronic stress and repeated mild stress effects, we used a chemogenetic approach. Specifically, the excitatory vector AAV-CaMKIIα-hM3D(Gq) was stereotaxically injected into the vSub. Activation of vSub neurons by clozapine N-oxide (CNO) suppressed stress-induced elevations in basal serum GC levels and reversed the increased immobility in the TST and FST, as well as the sociability deficits in CRST-treated young mice (Supplementary Fig. 2a–h). We further investigated whether activating vSub neurons could reverse stress-induced behavioral deficits in aged mice (14.5 months). Chemogenetic activation of vSub neurons with CNO in a manner similar to young mice suppressed the RST7d-induced elevation in serum GC levels and restored stress-induced behavioral deficits, including depressive-like behavior, reduced sociability and impaired social memory (Supplementary Fig. 2i–s).

**Fig. 2:**
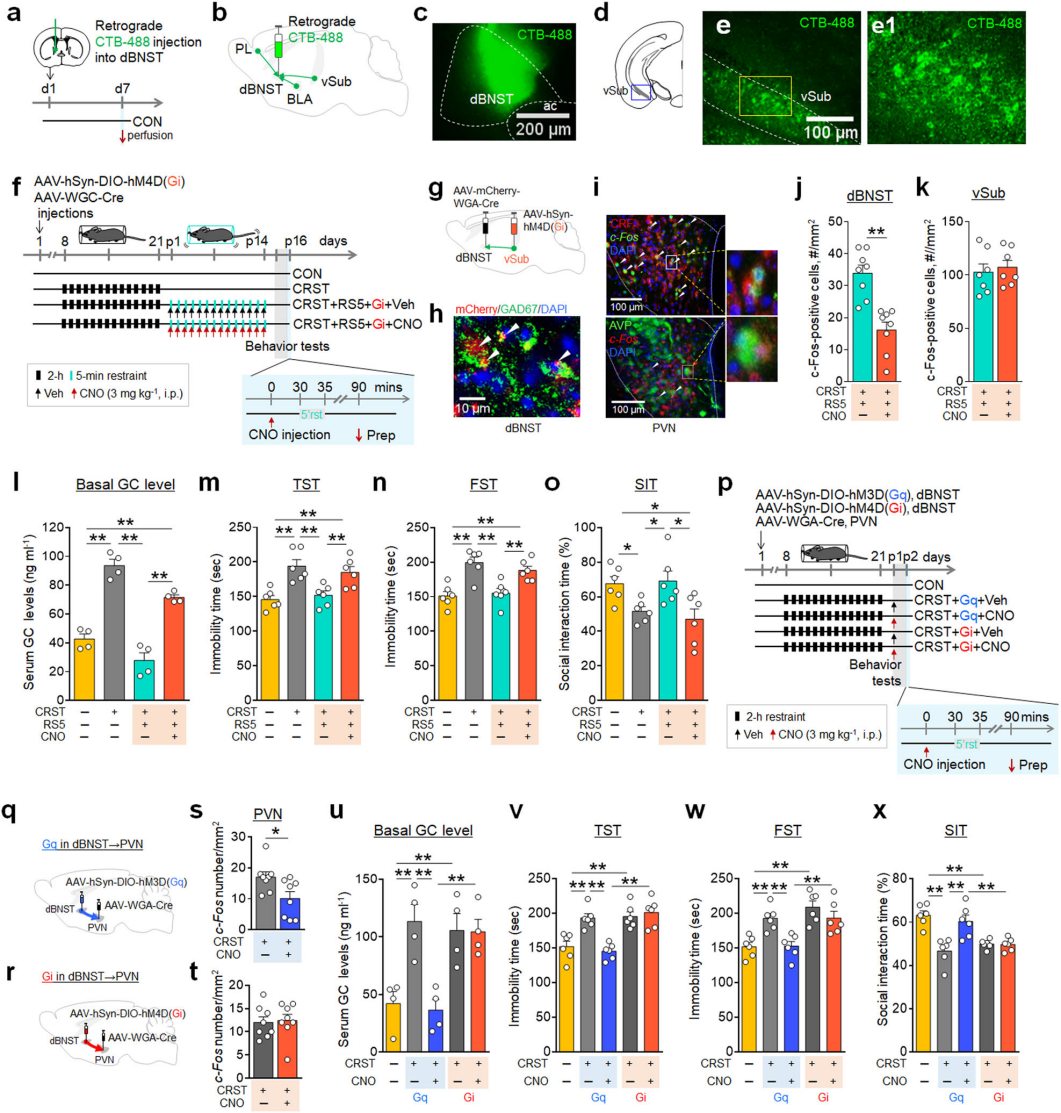
Chemogenetic inhibition of the vSub-to-dBNST circuit blocked repeated mild stress effects. **a**–**e** Experimental design (**a**). Diagram for the possible circuits projected to the dBNST (**b**). The retrograde tracer CTB488 was injected into the dBNST (**c**). Diagram for the location of vSub (**d**). Photomicrographs showing CTB488 localization in vSub neurons (**e**), with higher magnification of the box (**e1**). The details of this tracking experiment are presented in Supplementary Fig. 3. **f**–**k** Experimental design (**f**). Mice injected with AAV-hSyn-DIO-hM4D(Gi)-mCherry into the vSub and a retrograde AAV-WGA-Cre vector into the dBNST (**g**) were subjected to CRST, followed by repeated 5-min restraint (RS5), with CNO or vehicle (Veh) administration. Photomicrographs showing mCherry expression in the dBNST (**h**), and c-Fos expression in CRF- or AVP-positive cells (**i**) in the PVN of the indicated groups. Quantification of c-Fos expression in dBNST (**j**) and vSub (**k**). **l**–**o** Basal serum GC levels (**l**). Immobility time in the TST (**m**) and FST (**n**), as well as the social interaction (% time) with a target versus an empty in the SIT (**o**), for the indicated groups. (*n* = 6 animals per group). **p** Experimental design. Mice received an injection of AAV-hSyn-hM3D(Gq) or AAV-hSyn-hM4D(Gi) into the dBNST, followed by a retrograde Cre vector into PVN. They were then subjected to CRST. **q**–**t** Quantification of c-Fos expression in the PVN after CNO injection of the indicated groups (**q** and **s**, hM3D(Gq); **r** and **t**, hM4D(Gi)). **u**–**x** Basal serum GC levels (**u**), the immobility time in the TST (**v**) and FST (**w**) and social interaction (% time) in the SIT (**x**). Data are mean ± s.e.m. *,**, difference between indicated groups. *P < 0.05, **P < 0.01 (Student’s *t*-test; one-way ANOVA followed by Newman–Keuls post-hoc test). See Supplementary Data 4 for statistical details.

Given that the dBNST, primarily composed of inhibitory neurons, projects to the PVN^16,22^, we hypothesized that it mediates the vSub’s regulation of stress coping responses. To address this, we injected the retrograde tracer Alexa488-conjugated cholera toxin subunit B (CTB) into the dBNST, confirming that it receives projections from the vSub, as well as from the PL and BLA (Fig. 2a–e and Supplementary Fig. 3). To further test whether the vSub-to-dBNST circuit mediates repeated mild stress effects, we stereotaxically injected the inhibitory vector AAV-hSyn-DIO-hM4D(Gi) into the vSub and the retrograde AAV-WGA-Cre vector into the dBNST. Subsequent CNO injection in these mice inhibited c-Fos induction in the dBNST caused by repeated 5-min restraint. Furthermore, CNO-mediated inhibition of the vSub-to-dBNST pathway abolished the ability of repeated mild stress to suppress CRST-induced increases in basal GC levels, and to rescue stress-induced despair-like behavior and sociability deficits (Fig. 2f–o). Collectively, these results suggest that the vSub, specifically the vSub-to-dBNST circuit, serves as an upstream neural pathway regulating stress coping responses.

Next, we investigated the role of the dBNST-to-PVN pathway in regulating stress responses. The dBNST contains GABAergic neurons that project to the PVN^16,22,29^; activation of this dBNST-to-PVN pathway results in inhibitory inputs to the PVN. Mice were divided into two groups. Each group received a dBNST injection of either the excitatory vector AAV-hSyn-hM3D(Gq) or the inhibitory vector AAV-hSyn-hM4D(Gi). Subsequently, both groups received a WGA-Cre injection into the PVN, leading to the expression of hM3D(Gq) or hM4D(Gi) in the dBNST-to-PVN circuit. These mice were then subjected to CRST, as depicted (Fig. 2p). CNO injection in mice carrying hM3D(Gq) expression reduced c-Fos expression in the PVN. By contrast, CNO injection in mice carrying hM4D(Gi) expression did not change c-Fos expression levels in the PVN (Fig. 2q–t). Chemogenetic activation of the dBNST→ PVN circuit with CNO in CRST-treated mice expressing hM3D(Gq) reduced c-Fos expression in the PVN, suppressed the increased serum GC levels, decreased the increased immobility in the TST and FST, and improved the decreased sociability in the social interaction test (SIT) (Fig. 2u–x). By contrast, chemogenetic inhibition of the dBNST→ PVN circuit with CNO in CRST-treated mice expressing hM4D(Gi) had no effect on the increased serum GC levels, elevated immobility in the TST and FST, or impaired sociability in the SIT (Fig. 2u–x). These results suggest that the dBNST-to-PVN pathway plays a crucial role in regulating serum GC levels, and emotional and social behaviors, with its neural activity suppressed following CRST.

### Aged mice exhibited chronic stress-like gene expression signatures

**Fig. 3:**
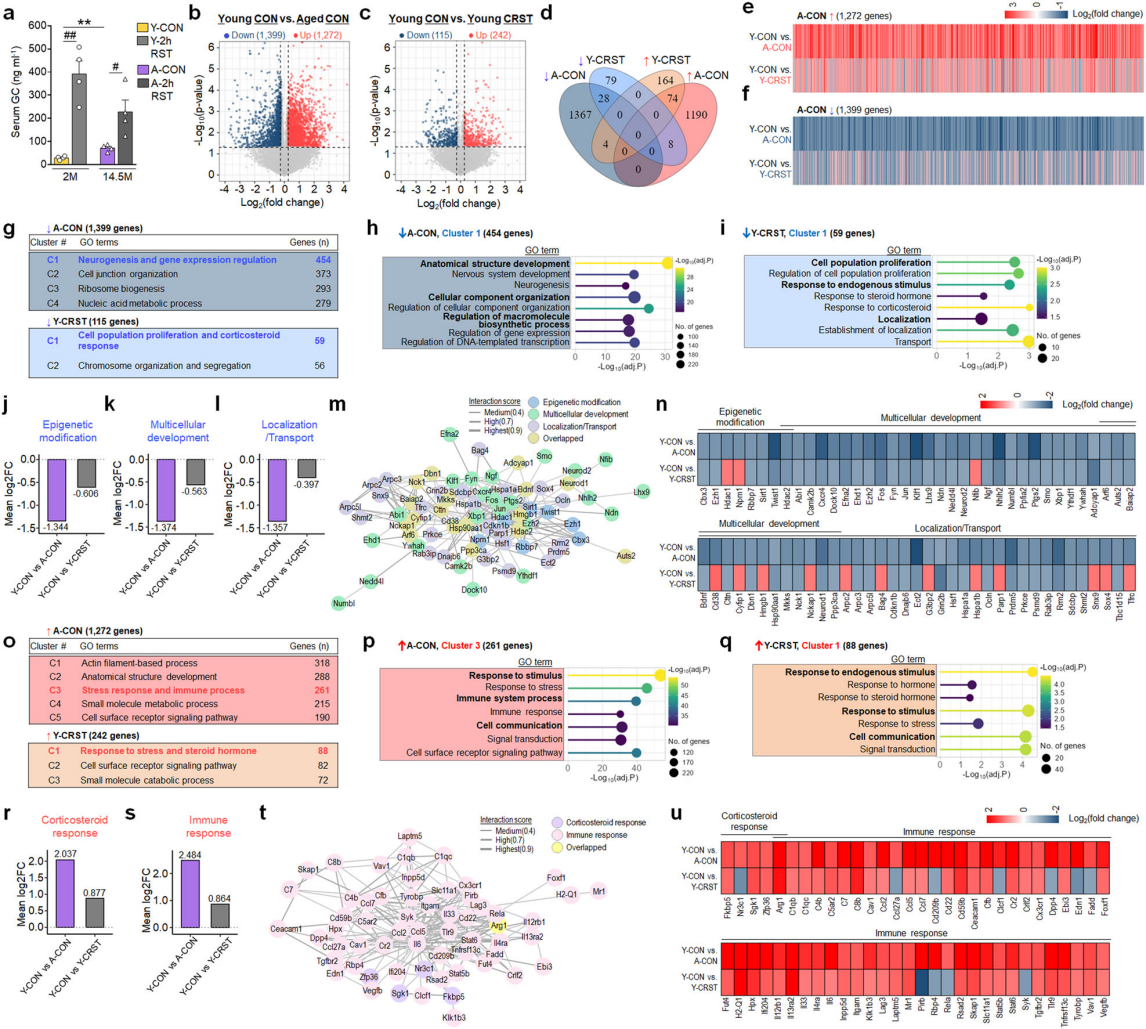
Aged mice exhibited vSub gene expression profiles indicative of a chronic stress signature. **a** Basal serum GC levels of young (2 M) and aged (14.5 M) mice, before and after 2-h restraint (*n* = 4 animals per group). **b**–**d** Volcano plots showing DEGs (≥1.15-fold, *P* ≤ 0.05) in aged versus young mice (**b**) and CRST-treated versus control young mice (**c**). Venn diagram illustrating the overlap of these DEGs (**d**). **e**, **f** Heatmaps showing concordant gene expression (upregulated 76.8%, downregulated 70.1%) between aged and CRST-treated young mice. **g**–**n** *K*-means clustering identified four functional clusters among aging-downregulated genes and two clusters within CRST-downregulated genes (**g**). Top BP terms for cluster 1 of aging-downregulated genes (**h**) and cluster 1 of CRST-downregulated genes (**i**) in young mice. GSVA-selected gene sets from aging-downregulated genes: epigenetic modification (**j**), multicellular development (**k**) and localization/transport (**l**), with their interaction network (**m**) and heatmaps (**n**) showing highly concordant gene expression patterns. **o**–**u** *K*-means clustering identified five functional clusters among aging-upregulated genes and three clusters for CRST-upregulated genes (**o**). Top BP terms for cluster 3 of aging-upregulated genes (**p**) and cluster 1 of CRST-upregulated genes (**q**) in young mice. GSVA-identified gene sets from aging-upregulated genes: corticosteroid response (**r**) and immune response (**s**), with their interaction network (**t**) and heatmaps (**u**) illustrating highly concordant gene expression patterns. Data are presented as mean ± s.e.m. (*n* = 4 animals per group). #, *P* < 0.05; **,##, *P* < 0.01 (Student’s *t*-test). See Supplementary Data 4 for statistical details.

Aged mice exhibited elevated baseline GC levels, but a diminished GC response to 2-h restraint (Fig. 3a). To understand the underlying mechanisms, we examined gene expression profiles in the vSub. RNA-seq analysis showed significant gene expression changes in aged mice (14.5 months) relative to young mice (2 months), identifying 1,272 upregulated and 1,399 downregulated genes (≥1.15-fold change, *P* ≤ 0.05). Intriguingly, CRST treatment in young mice induced similar, although less extensive, alterations in the vSub, affecting 242 upregulated and 115 downregulated genes (≥1.15-fold change, *P* ≤ 0.05). Among these, 74 upregulated and 28 downregulated genes were commonly altered in the vSub of both naive aged mice and CRST-treated young mice. Consistently, further analysis demonstrated a highly significant concordance in the overall patterns of up- and downregulated gene expression between aged mice and CRST-treated young mice (Fig. 3b–f and Supplementary Fig. 4a–l).

*K*-means clustering identified four clusters among the 1,399 genes downregulated by aging, including one associated with ‘neurogenesis and gene expression regulation’. By contrast, two clusters were found among the 115 genes downregulated by CRST in young mice, with one enriched for ‘cell population proliferation and corticosteroid response’. Gene set variation analysis (GSVA) of the aging-downregulated genes in cluster 1 revealed enriched, functionally interactive gene sets. These gene sets showed highly concordant expression patterns with the same genes in CRST-treated young mice, encompassing those involved in epigenetic modification (Cbx3, Ezh1, Hdac1, Hdac2 and Sirt1); multicellular development (Auts2, Bdnf, Fyn, Neurod1, Ngf, Nfib and Smo), and localization/ transport process (Arf6, Grin2b, Hsp90aa1, Hspa1a and Rab3ip) (Fig. 3g–n, Supplementary Fig. 4m–aa and Supplementary Data 1).

Similar *K*-means clustering revealed five functional groups from the 1,272 genes upregulated by aging, including one for ‘stress response and immune process’. By contrast, three clusters were identified among the 242 genes upregulated in CRST-treated young mice, notably with one enriched for ‘response to stress and steroid hormone’. GSVA of the aging-upregulated genes identified functionally interactive gene sets. These gene sets exhibited highly concordant expression patterns with the corresponding genes in CRST-treated young mice, including those for corticosteroid response (Nr3c1 (GR), Fkbp5 and Sgk1) and immune response (C1qb, Cx3cr1, IL33, IL6, Itgam(CD11b), Rela(NF-kB_p65), Syk, Tgfbr2, Tyrobp(DAP12) and Stat6) (Fig. 3o–u, Supplementary Fig. 4ab–ak and Supplementary Data 1).

### Repeated mild stress reversed stress-induced gene expression changes in aged mice

**Fig. 4:**
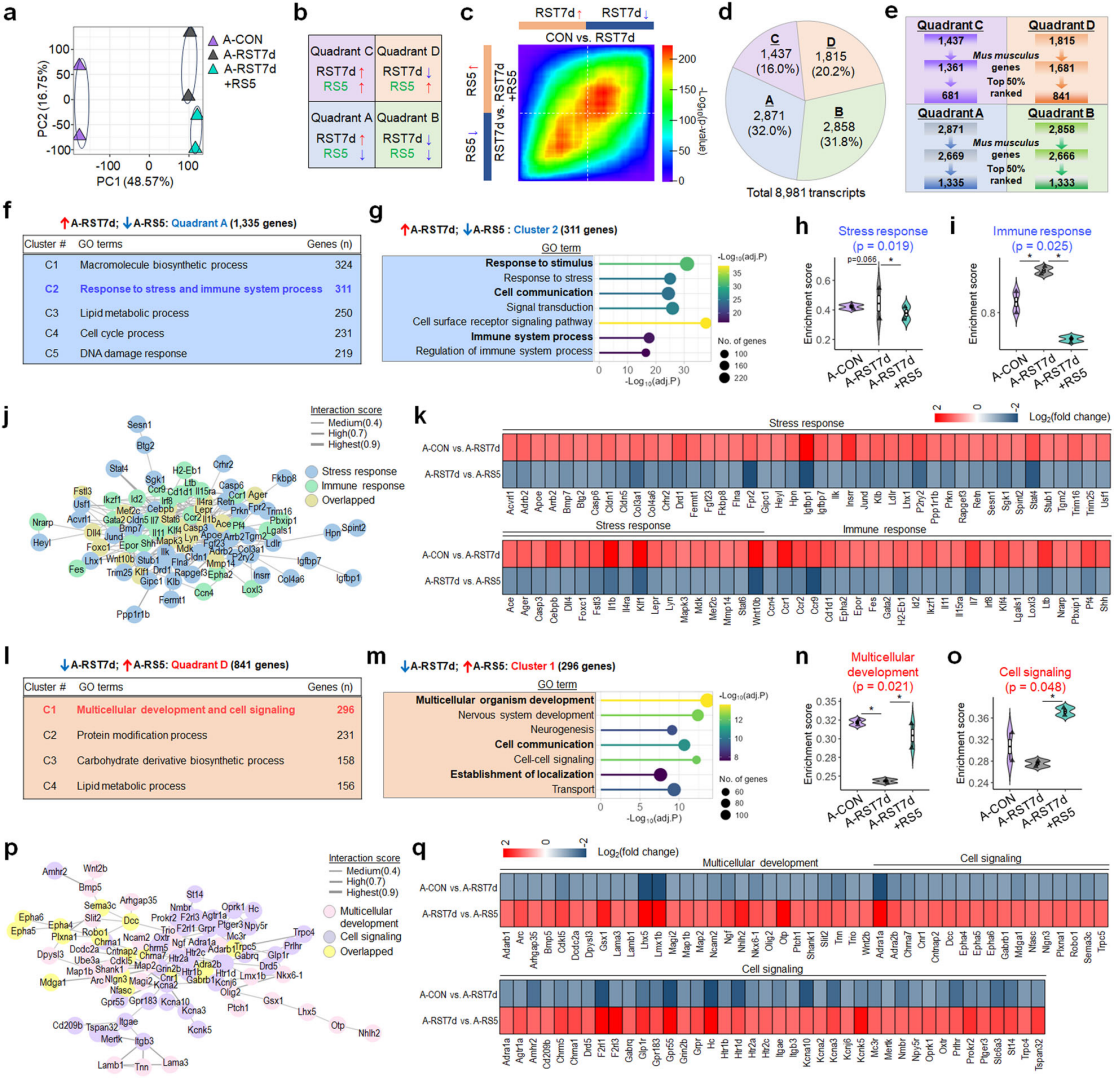
Repeated mild stress reversed stress-induced gene expression profiles in aged mice. **a**–**e** PCA (**a**) of differential gene expression variance in aged mice: PC1 (48.57% variance) primarily separated CON from RST7d, and PC2 (16.75% variance) distinguished RST7d from RST7d + RS5. Gene expression patterns in RRHO map (**b**). RRHO map visualizing differential gene expression in CON versus RST7d (*x* axis) and RST7d versus RST7d + RS5 (*y* axis) (**c**). The number (%) of identified genes within each quadrant (**d**). Flow charts (**e**) of selecting the top-50% ranked genes within each quadrant. **f**–**k** *K*-means clustering identified five functional clusters (**f**) among the RST7d-upregulated and RS5-downregulated genes in aged mice. Top BP terms for cluster 2 (**g**) of the RS5-downregulated genes. GSVA-selected gene sets identified among the RS5-downregulated genes across the CON, RST7d and RST7d + RS5 groups, including those for stress response (**h**) and immune response (**i**), with their interaction network (**j**) and heatmaps (**k**). **l**–**q** *K*-means clustering identified four functional groups (**l**) within the RST7d-downregulated genes. Top BP terms for cluster 1 (**m**) of the RS5-upregulated genes. GSVA-selected gene sets identified within the RS5-upregulated genes across the CON, RST7d and RST7d + RS5 groups, including those for multicellular development (**n**), and cell signaling (**o**), with their interaction network (**p**) and heatmaps (**q**). Violin plots are shown with overlaid boxplots displaying the mean and quartiles. The boxplot whiskers indicate 1.5 times the interquartile range from the first and third quartiles. Differences in GSVA scores among groups were assessed using one-way ANOVA followed by Tukey’s HSD post-hoc test. *P < 0.05.

We further investigated gene expression alterations by subchronic restraint stress (RST7d) in aged mice (14.5 months; CON) and their subsequent reversion following subsequent repeated mild stress. Principal component analysis (PCA) on RNA-seq reads indicated that principal component (PC)1 (48.57% of the total variance) differentiated the CON and RST7d groups, and PC2 (16.75% of the total variance) distinguished the RST7d from RST7d + RS5 groups (Fig. 4a).

RNA-seq analysis, coupled with RRHO, revealed that repeated mild stress reversed the expression of the top 50% of 1,335 genes upregulated (quadrant A) and 841 genes downregulated (quadrant D) following RST7d in aged mice (Fig. 4b–e). Serial *K*-means clustering of the 1,335 genes in quadrant A identified five functional clusters, including one involved in stress and immune responses. Gene Ontology (GO) enrichment analysis of this cluster identified biological process (BP) terms, including stress response (Casp3, Casp6, Cebpb, Crhr2, Fkbp8, Jund, Mapk3, Mef2c and Sgk1) and immune response (Cd1d1, Ccr1, Ccr2, H2-Eb1, Il1b, Il4ra, Il7, Irf8, Lgals1, Lyn and Stat6) (Fig. 4f–k and Supplementary Data 2).

Serial *K*-means clustering of the 841 genes in quadrant D revealed four functional clusters, including one involved in multicellular development and cell signaling. GSVA of this cluster revealed gene sets for multicellular development (Dcc (netrin-1 receptor), Dpysl3, Map1b, Ngf, Robo1, Shank1, Slit2 and Wnt2b) and cell signaling (Cnr1, Drd5, Gabrb1, Grin2b, Htr2a, Kcnj6, Mertk, Oprk1 and Oxtr) (Fig. 4l–q and Supplementary Data 2).

### Aged mice showed vastly divergent gene expression for stress responses than young mice, with minor commonalities

**Fig. 5:**
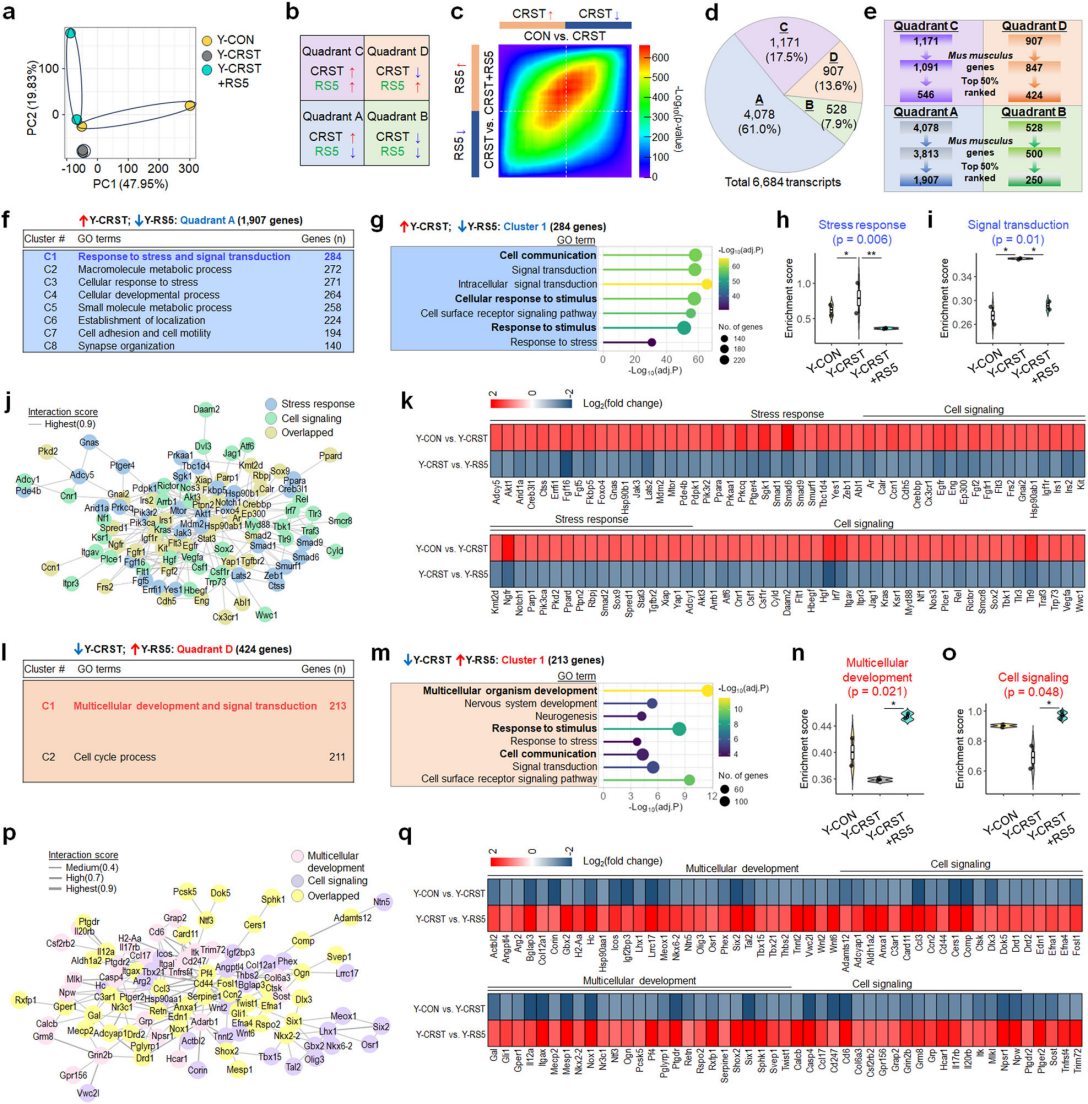
Repeated mild stress reversed stress-induced gene expression profiles in the vSub in young mice. **a**–**e** PCA (**a**) of DEGs in young mice, illustrating the variance between CON versus CRST (PC1; 47.95%) and CRST vs. RST7d + RS5 (PC2; 19.83%). Gene expression patterns in RRHO map (**b**). RRHO map visualizing differential gene expression in CON versus CRST (*x* axis) and CRST versus CRST + RS5 (*y* axis) (**c**). The number (%) of identified genes within each quadrant (**d**). Flow charts (**e**) of selecting the top-50% ranked genes within each quadrant. **f**–**k** *K*-means clustering identified eight functional groups within the CRST-upregulated and RS5-downregulated genes (quadrant A) in young mice (**f**). Top BP terms for cluster 1 of these RS5-downregulated genes (**g**). GSVA-selected gene sets identified among these genes across the CON versus CRST and CRST versus CRST + RS5 groups, including stress response (**h**) and signal transduction (**i**), with their interaction network (**j**) and heatmaps (**k**) showing gene expression changes. **l**–**q** *K*-means clustering revealed two functional groups within the CRST-downregulated and RS5-upregulated genes (quadrant D) in young mice (**l**). Top BP terms for cluster 1 of these RS5-upregulated genes (**m**). GSVA-selected gene sets identified within these genes across the CON versus CRST and CRST versus CRST + RS5 groups, including multicellular development (**n**) and cell signaling (**o**), with their interaction network (**p**) and heatmaps (**q**) showing gene expression alterations. Violin plots are shown with overlaid boxplots displaying the mean and quartiles. The boxplot whiskers indicate 1.5 times the interquartile range from the first and third quartiles. Differences in GSVA scores among groups were assessed using one-way ANOVA followed by Tukey’s HSD post-hoc test. *P < 0.05.

Next, we explored stress-induced gene expression changes and their reversal by repeated mild stress in young mice. PCA of RNA-seq data revealed distinct separation between groups: PC1 (47.95% of total variance) differentiated the CON and CRST groups, while PC2 (19.83% of total variance) distinguished the CRST from the CRST + RS5 groups (Fig. 5a).

RNA-seq analysis, combined with RRHO, revealed that repeated mild stress reversed the expression of the top 50% of the 1,907 genes upregulated and the 424 genes downregulated after CRST in young mice (Fig. 5b–e). *K*-means clustering of the 1,907 genes that were upregulated by CRST and whose altered expression was reversed by RS5 (quadrant A) identified eight functional clusters, including one for ‘response to stress and signaling transduction’. GSVA of this cluster revealed top BP terms, including stress response (Fkbp5, Sgk1, Pde4b, Stat3, Hsp90b1, Hsp90ab1, Parp1, Smad9, Tgfbr2 and Jak3) and immune response (Cnr1, Csf1, Csf1r, Irf7, Myd88, Rel(c-Rel), Tbk1, Tlr3, Tlr9 and Traf3) (Fig. 5f–k and Supplementary Data 3).

Among the 424 genes downregulated by CRST and whose altered expression was reversed by RS5 (quadrant D), *K*-means clustering revealed two functional clusters, one of which was associated with multicellular development and signal transduction. GO enrichment analysis of this cluster identified gene sets for multicellular development (Gli1, Hsp90aa1, Ntf3, Rspo2, Shox2, Six1, Twist1, Wnt2 and Wnt6) and cell signaling (Adcyap1, Drd1, Drd2, Gal, Grin2b, Mecp2, Nr3c1, Ptgdr and Sphk1) (Fig. 5l–q and Supplementary Data 3).

Next, we compared the gene expression profiles of aged mice (14.5 months), where expression was altered by RST7d and reversed by RS5, with those of young mice (2 months), where expression was similarly affected by CRST and reversed by RS5, to determine if expression changes overlapped in the same direction. A comparative analysis of gene expression profiles in quadrant A of aged and young mice identified 482 genes common to both age groups. The shared genes accounted for 16.8% of quadrant A genes in aged mice (11.8% in young mice) and were categorized into three functional clusters, including gene sets associated with response to stress and signal transduction (Apoe, Csf1, Fkbp5, Itgam, Ikbkb, Jak3, Pdgfb and Sgk1) (Supplementary Fig. 5a–e).

A similar analysis of gene expression profiles within quadrant D of aged (14.5 months) and young (2 months) mice revealed 99 genes shared between both age groups. These shared genes represented 5.5% of quadrant D genes in aged mice (10.9% in young mice) and were classified into three functional clusters, including gene sets involved in multicellular development and regulation to transport (Fkbp4, Grin2b (NR2b), Hsp90aa1, Mecp2 and Nr3c1 (GR)) (Supplementary Fig. 5f, g).

Collectively, these results suggest that, although the overall responses to subchronic stress in aged mice or chronic stress in young mice—and subsequent RS5 treatment—vary considerably across age groups, the gene sets associated with stress responses and multicellular development, particularly those in the GR–Mecp2–Fkbp5 pathway, are shared between both groups.

### The vSub GR-Fkbp5 responsive system mediated repeated mild stress effects

**Fig. 6:**
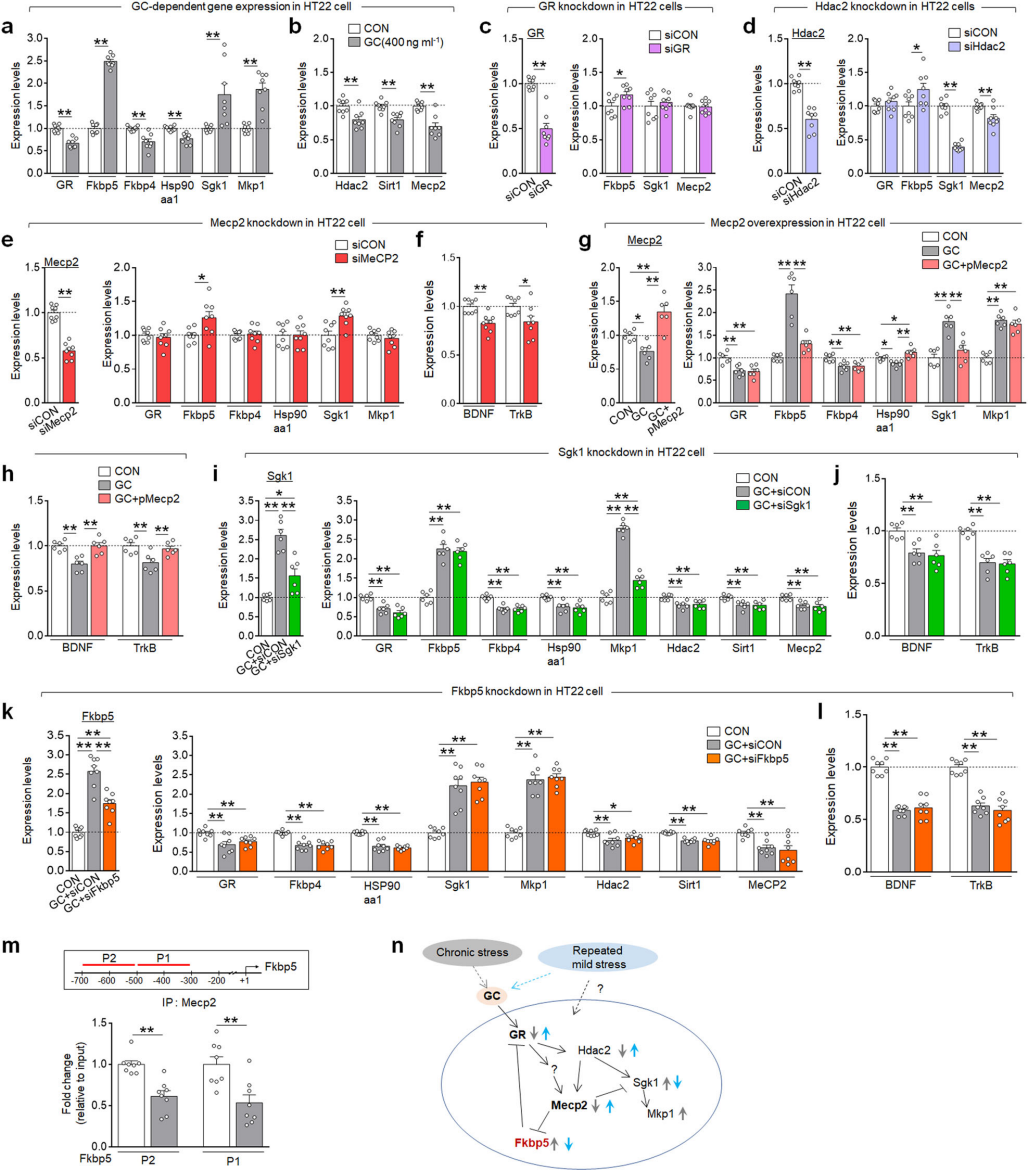
GC-dependent regulation of GR, Mecp2 and Fkbp5 gene expression and their associated pathways in HT22 cells. **a, b** GC effects on the expression of GR, Fkbp5, Fkbp4, Hsp90aa1, Sgk1, Mkp-1 (**a**), Hdac2, Sirt1 and Mecp2 (**b**) in HT22 cells (CON) and HT22 cells treated with GC (400 ng ml^-1^) for 24 h. **c** GR knockdown effects on the expression of GR, Fkbp5, Sgk1 and Mecp2 in HT22 cells treated with siRNA-control (siCON) or siRNA-GR (siGR) for 24 h. **d** Hdac2 knockdown effects on the expression of Hdac2, GR, Fkbp5, Sgk1 and Mecp2 in HT22 cells treated with siRNA-control or siRNA-Hdac2 (siHdac2) for 24 h. **e**, **f** Mecp2 knockdown effects on the expression of Mecp2, GR, Fkbp5, Fkbp4, Hsp90aa1, Sgk1, Mkp-1 (**e**), Bdnf and TrkB (**f**) in HT22 cells treated with siRNA-control or siRNA-Mecp2 (siMecp2) for 24 h. **g**, **h** Mecp2 overexpression effects on the expression of Mecp2, GR, Fkbp5, Fkbp4, Hsp90aa1, Sgk1, Mkp-1 (**g**), Bdnf and TrkB (**h**) in HT22 cells treated for 24 h with GC (400 ng/ml), or GC plus pCNS-D2-MeCP2 (pMeCP2). HT22 cells were transfected with pMeCP2 and treated with GC 24 h later. **i**, **j** Sgk1 knockdown effects on the expression of Sgk1, GR, Fkbp5, Fkbp4, Hsp90aa1, Mkp1, Hdac2, Sirt1, Mecp2 (**i**), Bdnf and TrkB (**j**) in HT22 cells treated for 24 h with GC plus siRNA-control, and GC plus siRNA-Sgk1. **k**, **l** Fkbp5 knockdown effects on the expression of Fkbp5, GR, Fkbp4, Hsp90aa1, Sgk1, Mkp1, Hdac2, Sirt1, Mecp2 (**k**), Bdnf and TrkB (**l**) in HT22 cells treated for 24 h with GC or GC plus siRNA-Fkbp5. **m** ChIP assay showing Mecp2 binding to the proximal promoter (P1 and P2 regions) of the Fkbp5 gene in HT22 cells treated with GC (400 ng/ml) for 24 h. **n** A summary of GC-induced changes in the expression of GR, Hdac2, Sgk1, Mkp-1, Mecp2 and Fkbp5 and their functional pathway. Data are presented as mean ± s.e.m. (*n* = 2–4 wells per group; 2–3 PCR repeats per group). *P < 0.05; **P < 0.01 (Student’s *t*-test; one-way ANOVA followed by the Newman–Keuls post-hoc test). See Supplementary Data 4 for statistical details.

Next, we investigated the regulatory mechanisms of GR-responsive genes and their downstream targets in vitro and in vivo. GC treatment in HT22 neuronal cells downregulated GR, Fkbp4, Hsp90aa1, Hdac2, Sirt1 and Mecp2, while upregulating Fkbp5, Sgk1 and Mkp-1. siRNA-mediated GR knockdown increased Fkbp5, but had no effect on Sgk1 and Mecp2. Hdac2 knockdown led to the suppression of Sgk1 and Mecp2, while inducing Fkbp5 (Fig. 6a–d). Mecp2 knockdown increased Fkbp5 and Sgk1, while decreasing Bdnf and TrkB. Conversely, Mecp2 overexpression suppressed GC-induced Fkbp5 and Sgk1 expression, but enhanced the GC-induced downregulation of Hsp90aa1, Bdnf and TrkB (Fig. 6e–h). Sgk1 knockdown reduced GC-dependent Mkp1 expression. Fkbp5 knockdown had no effect on GC-dependent changes in GR, Fkbp4, Hsp90aa1, Sgk1, Mkp-1, Hdac2, Sirt1, Mecp2, Bdnf and TrkB expression (Fig. 6i-l). ChIP assay indicated that GC treatment decreased MeCP2 binding to the proximal promoter of Fkbp5 (Fig. 6m), suggesting that MeCP2 is an upstream regulator of Fkbp5 expression. Collectively, these results suggest that GR–Mecp2–Fkbp5 acts as a key functional module in GC-activated cellular responses (Fig. 6n).

**Fig. 7:**
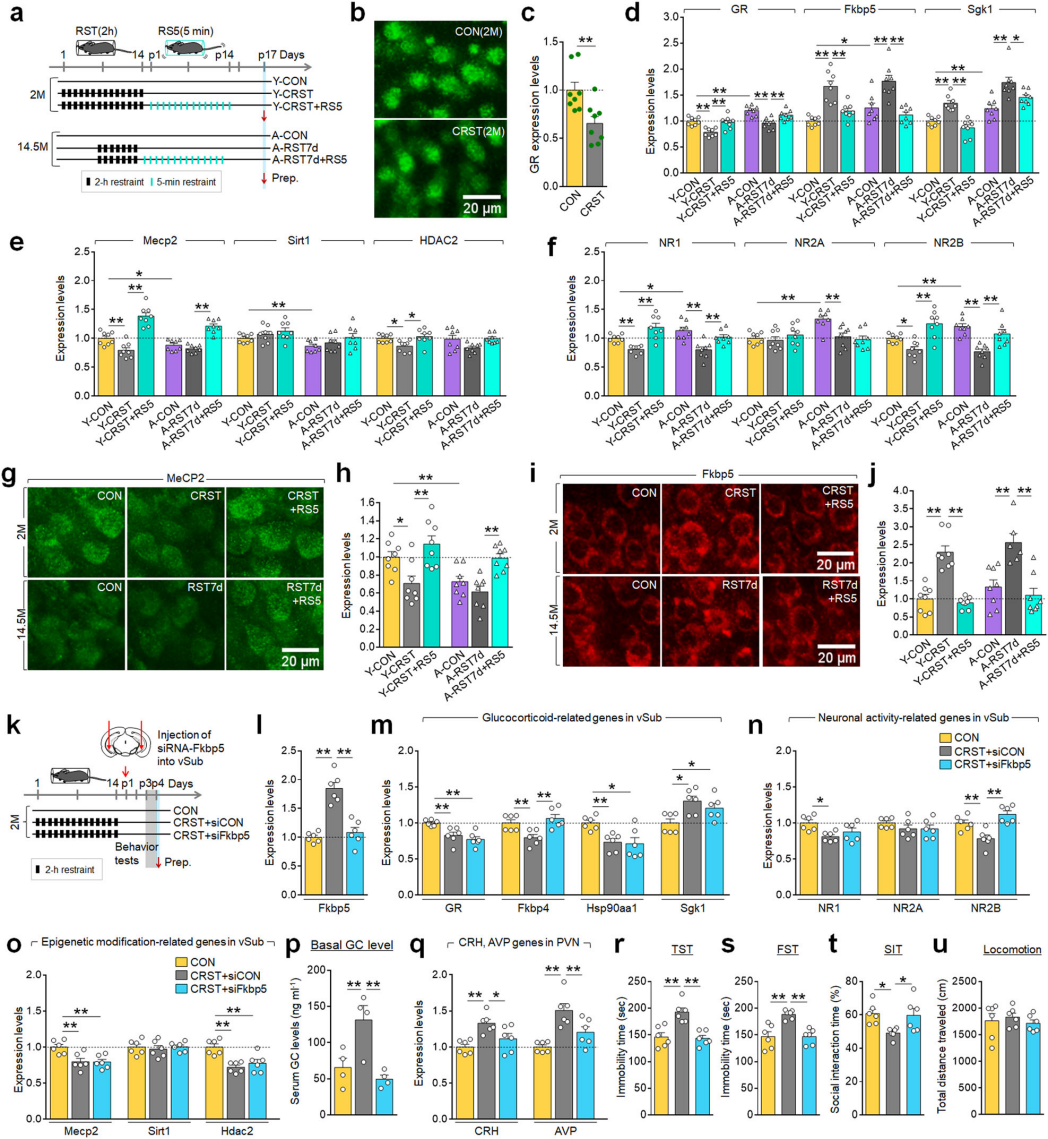
Repeated mild stress restored altered Fkbp5 function in the vSub of young and aged mice. **a** Experimental design. Mice (2 M) and aged mice (14.5 M) were subjected to CRST or RST7d, respectively. Subsequently, they were treated with repeated mild stress as illustrated. **b**, **c** Photomicrographs showing CRST-induced GR downregulation in the vSub of young mice (**b**). Quantification levels (**c**). **d**–**f** Transcript levels of GR, Fkbp5 and Sgk1 (**d**); Mecp2, Sirt1 and Hdac2 (**e**); and NR1, NR2A and NR2B (**f**) in the vSub of the indicated groups. **g**–**j** Photomicrographs and quantification of MeCP2 (**g** and **h**) and Fkbp5 (**i** and **j**) expression levels in the vSub of the indicated groups (*n* = 4 per group). **k** Experimental design. Mice (2 M) were subjected to CRST, followed by siRNA-Fkbp5 or siRNA-control injection into the vSub on both sides. **l**–**q** Transcript levels of Fkbp5 (**l**), GR, Fkbp4, Hsp90aa1, Sgk1 (**m**), NR1, NR2A, NR2B (**n**), Mecp2, Sirt1 and Hdac2 (**o**) in the vSub. Basal serum GC levels (**p**). CRH and AVP expression (**q**) in the PVN of the indicated groups (*n* = 4 animals; 3 PCR repeats per group). **r**–**u** The immobility time in the TST (**r**) and FST (**s**). Social interaction (% time) (**t**) and locomotor activity (**u**) in the SIT for the indicated groups (*n* = 6 animals per group). Data are mean ± s.em. *P < 0.05, **P < 0.01 (Student’s *t*-test; one-way ANOVA followed by Newman–Keuls post-hoc test). See Supplementary Data 4 for statistical details.

We examined the expression of GR-responsive genes in the vSub of young and aged mice (14.5 months). Immunohistochemistry and reverse-transcription PCR analyses revealed that CRST decreased GR expression in the vSub in young mice. Aged mice exhibited increased expression of GR, as well as Fkbp5, Sgk1, NR1, NR2A and NR2B, and decreased expression of Sirt1 and Mecp2, compared with young mice. Subchronic stress (RST7d) in aged mice further enhanced Fkbp5 and Sgk1 expression, while decreasing GR, Mecp2, NR1 and NR2B, with these altered expressions being comparable to those induced by CRST in young mice. Furthermore, repeated treatment with daily 5-min restraint similarly reversed these stress-induced gene expression alterations in both young and aged mice (Fig. 7a–f).

Immunohistochemical analysis showed that RST7d treatment in aged mice (14.5 months) resulted in a reduction in MeCP2 expression and an increase in Fkbp5 expression within the vSub. These effects were comparable to those induced by CRST in young mice. Stress-or aging-dependent alterations in MeCP2 and Fkbp5 expression were primarily detected in neurons with large cell bodies, presumably pyramidal neurons. By contrast, repeated 5-min restraint treatment reversed the stress-induced changes in the expression of these factors in both young and aged mice (Fig. 7g–j).

We investigated the functional significance of the GR–Mecp2–Fkbp5 pathway in the vSub. siRNA-mediated Fkbp5 knockdown within the vSub reversed the CRST-induced decreases in Fkbp4 and NR2B expression, while having no significant effect on GR, Hsp90aa1, Sgk1, NR1, NR2A, Mecp2, sirt1 and Hdac2 (Fig. 7k–o). The Fkbp5 knockdown also suppressed the CRST-induced increases in basal serum GC levels and CRH and AVP expression in the PVN, and also restored the CRST-induced depressive-like behaviors and sociability deficits (Fig. 7p–u). Similarly, Fkbp5 knockdown within the vSub in aged mice (14.5 months) suppressed the RST7d-induced CRH and AVP expression in the PVN. Furthermore, Fkbp5 knockdown reversed stress-induced despair-like behavior and impaired sociability (Supplementary Fig. 6). These results suggest that vSub Fkbp5 is a key player in regulating stress-induced dysfunction of the HPA axis response and emotional and social behaviors.

### Repeated mild stress reversed aging- and stress-induced behavioral changes

**Fig. 8:**
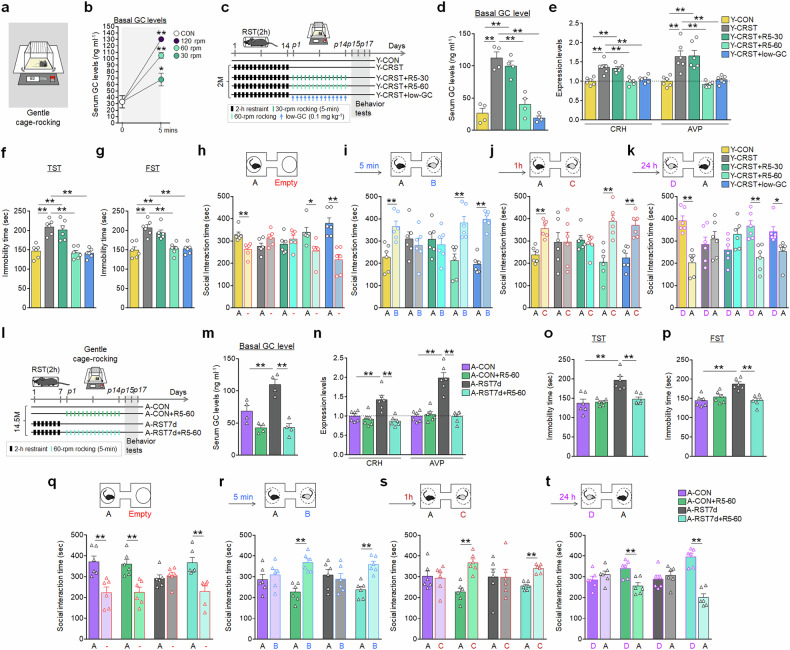
Repeated gentle-rocking stress reversed stress-induced emotional and social behavioral deficits in aged mice. **a**, **b** An orbital shaker for gentle rocking (**a**). Serum GC levels (**b**) after 5-min gentle rocking at varying speeds (*n* = 6 animals per group). **c** Experimental design. Young mice (2 M) subjected to CRST were treated with repeated 5-min gentle rocking (designated R5) at 30 rpm or 60 rpm for 14 days, or treated with low-dose GC (0.1 mg/kg/day) for 14 days (*n* = 6 animals per group). **d**, **e** Basal serum GC levels (**d**). Expression levels of CRH and AVP in the PVN (**e**) for the indicated groups. **f**–**k** Immobility time in the TST (**f**) and FST (**g**). Social behavior test: interaction time (**h**) with a target versus an empty in the SIT; and interaction time with familiar versus novel targets in the SMT at 5 min (**i**), 1 h (**j**) and 24 h (**k**) post-SIT for the indicated groups. **l** Experimental design. Aged mice (14.5 M) underwent 14 days of 5-min daily gentle rocking at 60 rpm. A separate group of aged mice undergoing subchronic stress (RST7d) were subjected to the same 14-day gentle rocking treatment (*n* = 6 animals per group). **m**, **n** Basal serum GC levels (**m**). Transcript levels (**n**) of CRH and AVP in the PVN for the indicated groups. **o**–**t** The immobility time in the TST (**o**) and FST (**p**). Social behavior test: interaction time (**q**) with a target versus an empty in the SIT; and interaction time with familiar versus novel targets in the SMT at 5 min (**r**), 1 h (**s**) and 24 h (**t**) post-SIT for the indicated groups. Data are mean ± s.e.m. *P < 0.05, **P < 0.01 (Student’s *t*-test; one-way ANOVA followed by Newman–Keuls post-hoc test). See Supplementary Data 4 for statistical details.

We further investigated whether mild, restraint-unrelated stressors, such as gentle rocking, could produce similar therapeutic effects. Notably, a 5-min session of gentle cage-rocking at 60 rpm increased serum GC levels, reaching 105.1 ng/ml in young mice (Fig. 8a, b). Repeated daily gentle rocking in young mice effectively suppressed the CRST-induced increases in basal GC levels, CRH and AVP expression within the PVN. This treatment also reversed CRST-induced depressive-like behavior and impairments in social behaviors (Fig. 8c–k). The physiological and behavioral rescues by gentle rocking were similar to those achieved with repeated low-dose GC treatment (0.1 mg/kg/day) (Fig. 8c–k).

Repeated daily 5-min gentle-rocking in aged mice (14.5 months) also reversed the RST7d-induced increases in basal serum GC levels and CRH and APV expression in the PVN (Fig. 8l–n). Aged and young mice showed no motor or sensory deficits before or after RST7d or CRST treatment, or following repeated mild stress (Supplementary Fig. 7). This treatment also reversed the RST7d-induced emotional and social behavioral impairments (Fig. 8o–t), and restored social memory deficits observed in normal aged mice (Fig. 8q–t), demonstrating its broad therapeutic potential.

CRST-treated young mice (2 months) and RST7d-treated aged mice (14.5 months) both exhibited decreased expression of GR, Fkbp4, Mecp2, NR1, NR2B, Bdnf and TrkB expression in the vSub, while Fkbp5 and Sgk1 expression was elevated. By contrast, repeated daily gentle rocking reversed these stress-induced alterations in both young and in aged mice (Supplementary Fig. 8).

## Discussion

The present study demonstrated that aged mice display physiological and molecular changes similar to those observed in young mice subjected to CRST. In particular, CRST in young mice (2 months) resulted in elevated GC levels, as well as increased expression of CRH and AVP in the PVN (Fig. 1), indicating overall dysregulation of the HPA axis in these mice. Similarly, aged mice (14.5 months) displayed HPA axis dysfunction, characterized by elevated basal GC levels and a blunted GC response to acute stress (2-h restraint) (Figs. 1m–p and 3a). Notably, the gene expression profile in the vSub of aged mice closely resembled the molecular changes induced by CRST in young mice (Figs. 3–5 and 7). This convergence included the downregulation of Mecp2, Fkbp4, NR1 and NR2B, along with the upregulation of Fkbp5 and Sgk1, genes tightly associated with GC-dependent pathways (Figs. 3–5 and 7 and Supplementary Figs. 4 and 5). Although the present study was limited to male mice, our results indicate that the aging brain exhibits molecular and physiological signatures characteristic of chronic stress, and that these chronic stress-like signatures in aged mice are probably driven by the cumulative actions of elevated GCs over time. Such age-dependent alterations in the GC-dependent pathway may place aged mice in a subchronic stress-like state and make them susceptible to upcoming stress.

Our activity-dependent c-Fos expression mapping identified several hippocampal subregions, including the dorsal and ventral CA1, CA3 and dentate gyrus, were strongly activated in response to short-term restraint in both young and aged mice (Supplementary Fig. 1). Among these subregions, the vSub functions as a principal hippocampal output, projecting to cortico-limbic regions such as the prefrontal cortex, nucleus accumbens and BLA, as well as to the dBNST, which in turn regulates the PVN^16,20,30–32^. Because the dBNST is composed mainly of GABAergic neurons^16,32^, its activation effectively inhibits the PVN activity. Indeed, chemogenetic stimulation of vSub neurons, particularly the vSub-to-dBNST circuit, using a DREADD system, suppressed the stress-induced enhanced basal GC levels, as well as emotional and social behavioral deficits (Fig. 2 and Supplementary Fig. 2). Thus, the vSub neuroanatomically posits as an upstream brain region that regulates both HPA axis activity and emotional and social behaviors.

Furthermore, vSub neurons utilize the GR–MeCP2–Fkbp5 signaling pathway as a cellular functional module. Normally, GC effects are initiated by binding to GC receptors (GRs). GC-activated GRs enter the nucleus, where they induce transcriptional activation or repression of targets genes^33,34^. Fkbp5 acts as a critical regulator of GR nuclear translocation^35,36^; its own expression is controlled by GC/GR signaling and can also be modulated by factors such as oxidative stress and inflammation^35–38^. GC/GR also negatively regulates MeCP2 expression, and MeCP2, in turn, acts as an upstream regulator for Fkbp5 expression in HT22 cells (Fig. 6). Consistently, chronic stress downregulates MeCP2 expression, along with upregulating Fkbp5 expression in the vSub of mice (Fig. 7 and Supplementary Fig. 6). Thus, GR, MeCP2 and Fkbp5 are closely interconnected at cellular and transcriptional levels.

The GR–MeCP2–Fkbp5 signaling module in the vSub appears to play a dual role: functioning as a molecular sensor that detects physiological stress states, and as a molecular effector that dynamically regulates GC-dependent gene expression to reshape vSub neuronal functions. As a molecular sensor, the vSub is highly responsive to GC, initiating activation of the GR–MeCP2–Fkbp5 pathway. CRST upregulated Fkbp5 expression in the vSub, whereas the Fkbp5 knockdown within the vSub was sufficient to restore stress-induced increases in basal GC levels and associated behavioral deficits in young and aged mice (Fig. 7 and Supplementary Fig. 6). Repeated mild stress downregulated Fkbp5 expression, while upregulating GR and MeCP2 expression in the vSub (Figs. 7 and 8), thereby intervening in the vicious cycle of the GR–MeCP2–Fkbp5 signaling activity in young and aged mice (Fig. 8). Similar effects were produced following repeated low-dose GC treatment. Collectively, these results indicate that the GC/GR-regulated MeCP2/Fkbp5 system in the vSub functions as a molecular mediator for both chronic stress and repeated mild stress effects in young and aged mice. Although Sgk1 does not appear to be a core component of the GR–MeCP2–Fkbp5 signaling module, its expression was notably elevated in the vSub of aged mice and further increased following CRST in young mice and RST7d in aged mice (Fig. 7). Interestingly, repeated mild stress (RS5) reversed the stress-induced upregulation of Sgk1 in the vSub of young and aged mice (Fig. 7). Given that hippocampal-specific knockdown of Sgk1 or pharmacological inhibition of Sgk1 conferred resilience to stress-induced behavior deficits^39,40^, it will be important to further investigate the role of Sgk1 in the vSub in modulating stress responses in aged mice.

As a molecular effector, the GR–MeCP2–Fkbp5 signaling module modulates the expression of NMDA receptor subunits, thereby influencing synaptic plasticity and neuronal activity within the vSub. Chronic stress decreased NR1 and NR2B expression in the vSub of CRST-treated young mice, as well as RST7d-treated aged mice. Conversely, repeated mild stress restored the expression of these NMDA receptor subunits (Fig. 7). These results suggest that chronic stress suppresses NMDA receptor-dependent neural activity in vSub neurons, whereas repeated mild stress reverses this suppression and restores normal activity.

Gentle rocking at 60 rpm for 5 min increased serum GC levels, peaking at 105.1 ng/ml (Fig. 8a, b), which is comparable to the GC levels (97.4 ng/ml) induced by 5-min daily restraint^18^. In fact, repeated treatment with daily low-dose GC (0.1 mg/kg/day) alone replicated the physiological and behavioral effects of repeated short-term mild stress (Fig. 8c–k). These data indicate that the therapeutic effects of repeated mild stress are probably mediated by low levels of GC surges induced by mild stress. The therapeutic effects of repeated mild stress or low-dose GC treatment raise an intriguing question of whether the physiological effects of repeated mild stress or low-dose GC treatment involve mechanisms related to the natural circadian rhythm of GC release. In healthy individuals, natural circadian GC rhythms typically peak in the early morning and reach their nadir around midnight^41^. Notably, the GC levels observed following 5-min restraint or gentle rocking (Figs. 1 and 8) align with these peak GC concentrations during the normal circadian cycle^42,43^. It is possible that the molecular stress signatures observed in the aging brain stem from a decline in circadian GC rhythms that normally mitigates or reset daily stress effects. Repeated mild stress therapy might, therefore, serve to reinstate this regulatory function. Further investigation is warranted to elucidate the detailed mechanisms and the potential of repeated mild stress therapy in modulating neurobehavioral responses, as well as other stress-related and stress-aggravated brain disorders.

## Supplementary information

Supplementary Information

Supplementary Data 1. DEGs in Y-CON versus A-CON and Y-CON versus Y-CRST_RV_2025_11_11a.

Supplementary Data 2. DEGs_RST7d_RS5 effects_aged mice_RV_2025_11_11a.

Supplementary Data 3. DEGs_CRST_RS5 effects_Young mice_RV_2025_11_11a.

nr-reporting-summary

Supplementary Data 4. Details of Statistical Analysis_RV_2025_11_11aa.

## Data Availability

All data collected or analyzed in this study are available within the Article and its Supplementary Information. Further information and requests for resources and reagents should be directed to and will be fulfilled by the corresponding author.
