# Supplementary material for: Repeated treatment with short-term mild stress reverses aging- and stress-induced emotional and social behavioral deficits
Source: Exp Mol Med. 2026 Feb 12;58(2):519–32. doi: 10.1038/s12276-026-01641-2 (PMC12993044; doi:10.1038/s12276-026-01641-2)
Supplement: Supplementary file 1 — Supplementary Information [file 12276_2026_1641_MOESM1_ESM.pdf]

SUPPLEMENTARY INFORMATION

Repeated treatment with short-term mild stress reverses aging- and stress-induced emotional and social behavioral deficits

Lee, E.H. et al.

Corresponding authors: Dr. EH Lee (e-mail: ehlee23@ewha.ac.kr), and
Dr. PL Han (e-mail: plhan@ewha.ac.kr)

Illustrations: **Supplementary Figures 1, 2, 3, 4, 5, 6, 7, and 8.**

Supplementary Detailed Materials and Methods

Supplementary Figures (1, 2, 3, 4, 5, 6, 7, and 8)

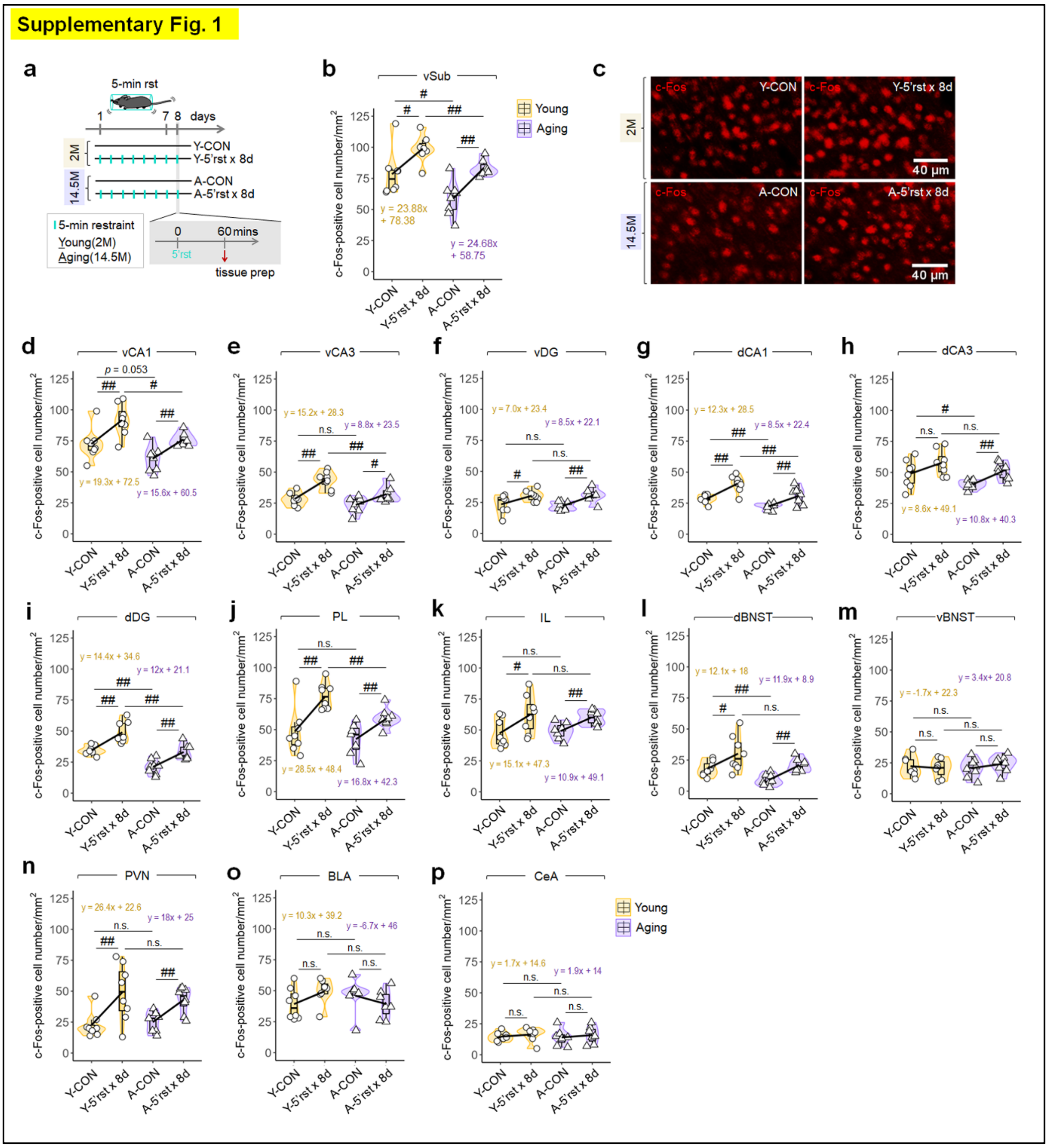

Supplementary Fig. 1. Repeated mild stress induced similar c-Fos expression patterns across various brain regions in both young and aged mice.

a, Experimental design. Mice (2 months; Y) and aged mice (14.5 months; A) were treated with a repeated mild stress protocol (daily 5-min restraint sessions for 8 days; 5’rst x 8d), and then sacrificed 60 min after the last 5-min restraint.

b–p, Repeated 5-min restraint increased c-Fos expression across brain regions of young and aged mice. Quantification of c-Fos expression levels in the ventral subiculum (vSub) (**b**), ventral Cornu Ammonis 1 (vCA1) (**d**), ventral CA3 (vCA3) (**e**), ventral dentate gyrus (vDG) (**f**), dorsal CA1 (dCA1) (**g**), dorsal CA3 (dCA3) (**h**), dorsal

DG (dDG) (**i**), prelimbic cortex (PL) (**j**), infralimbic cortex (IL) (**k**), dorsal bed nucleus of the stria terminalis (dBNST) (**l**), ventral BNST (vBNST) (**m**), hypothalamic paraventricular nucleus (PVN) (**n**), basolateral nucleus of the amygdala (BLA) (**o**), and central nucleus of the amygdala (CeA) (**p**). Photomicrographs showing c-Fos expression in the vSub (**c**) of young control (Y-CON), aged control (A-CON), young mice treated with repeated mild stress (Y-5'rst x 8d), and aged mice treated with repeated mild stress (A-5'rst x 8d) (*n* = 4 animals per group).

Data are mean ± SEM. $^{\#,\#\#}$, difference between indicated groups; $^{\#}$, $p < 0.05$; $^{\#\#}$, $p < 0.01$ (Student's t-test). See Supplementary Data 4 for statistical details.

Supplementary Fig. 2

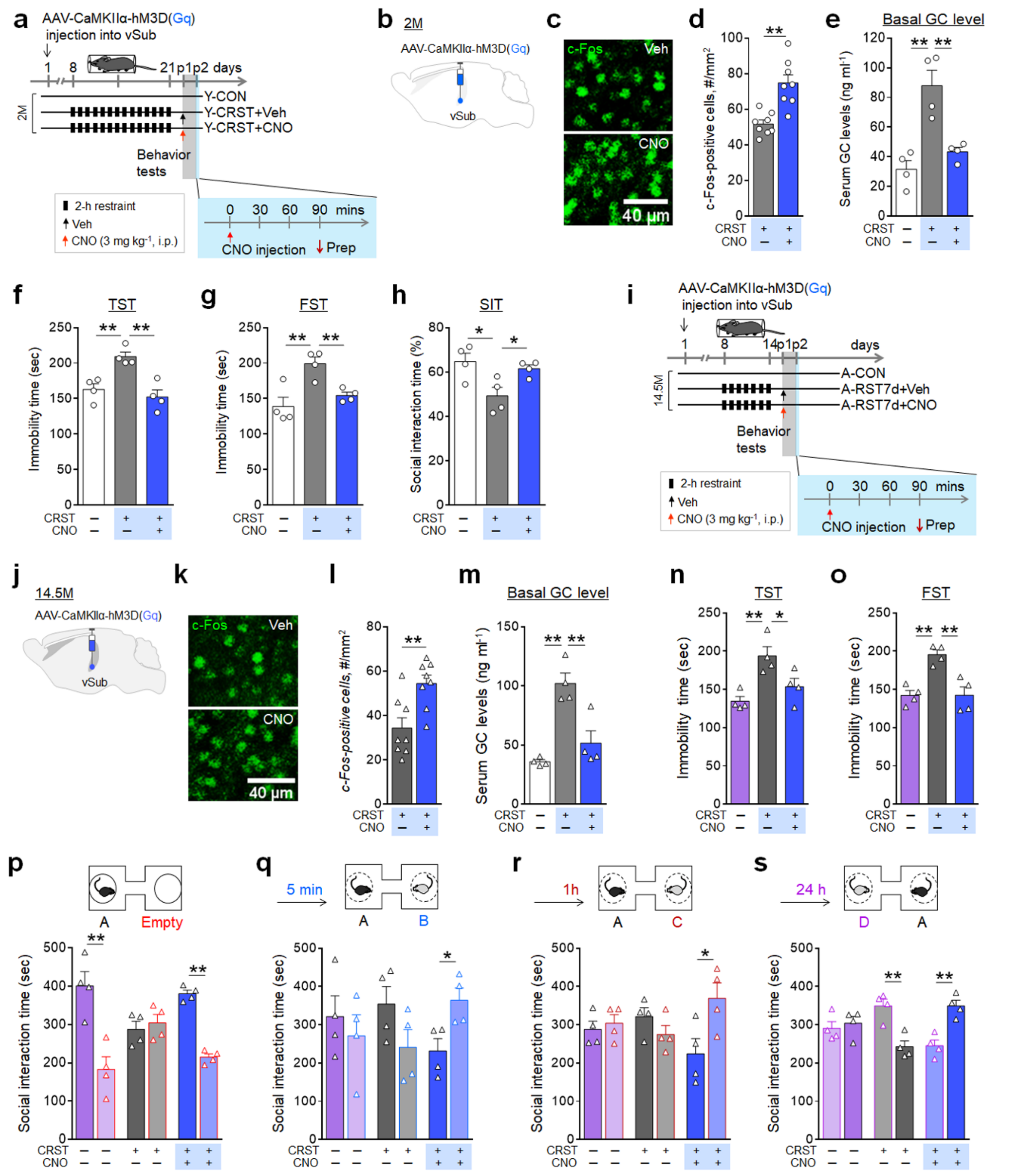

Supplementary Fig. 2. Chemogenetic activation of vSub neurons abolished neurophysiological and behavioral effects of repeated mild stress.

a–d, Experimental design (**a**). Mice injected with AAV-CaMKIIα-hM3D(Gq) into the vSub (**b**) were subjected to CRST. Behavioral tests were conducted one hour after vehicle (Veh) or clozapine-N-oxide (CNO) injection. On next day, mice were sacrificed after Veh or CNO injection. Photomicrographs showing c-Fos expression in the vSub after CNO or Veh injection (**c**). Quantification levels (**d**) (n = 4 per group). CNO dose, 3.0 mg kg^{-1} per injection (i.p.).

e–**h**, Basal serum GC levels (**e**). The immobility time in the TST (**f**) and FST (**g**). The interaction time with a target versus an empty in the SIT (**h**) for the indicated groups (*n* = 4 animals per group).

i-l, Experimental design (**i**). Aged mice (14.5 M) injected with AAV-CaMKIIα-hM3D(Gq) into the vSub (**j**) were subjected to RST7d. Behavioral tests were conducted one hour after CNO or Veh administration. Photomicrographs showing c-Fos expression in the vSub after CNO or Veh injection (**k**). Quantification levels (**l**) (*n* = 4 per group). CNO dose, 3.0 mg kg^{-1} per injection (i.p.).

m–**s**, Basal serum GC levels (**m**). The immobility time in the TST (**n**) and FST (**o**). The interaction time (**p**) with a target versus an empty in the SIT, and the interaction time with familiar versus novel targets in the SMT at 5 min (**q**), 1 h (**r**), and 24 h (**s**) post-SIT for the indicated groups (*n* = 4 animals per group).

Data are mean ± SEM. *,**, difference between indicated groups. *, $p < 0.05$; **, $p < 0.01$ (Student's t-test; One-way ANOVA followed by Newman-Keuls post-hoc test). See Supplementary Data 4 for statistical details.

Supplementary Fig. 3

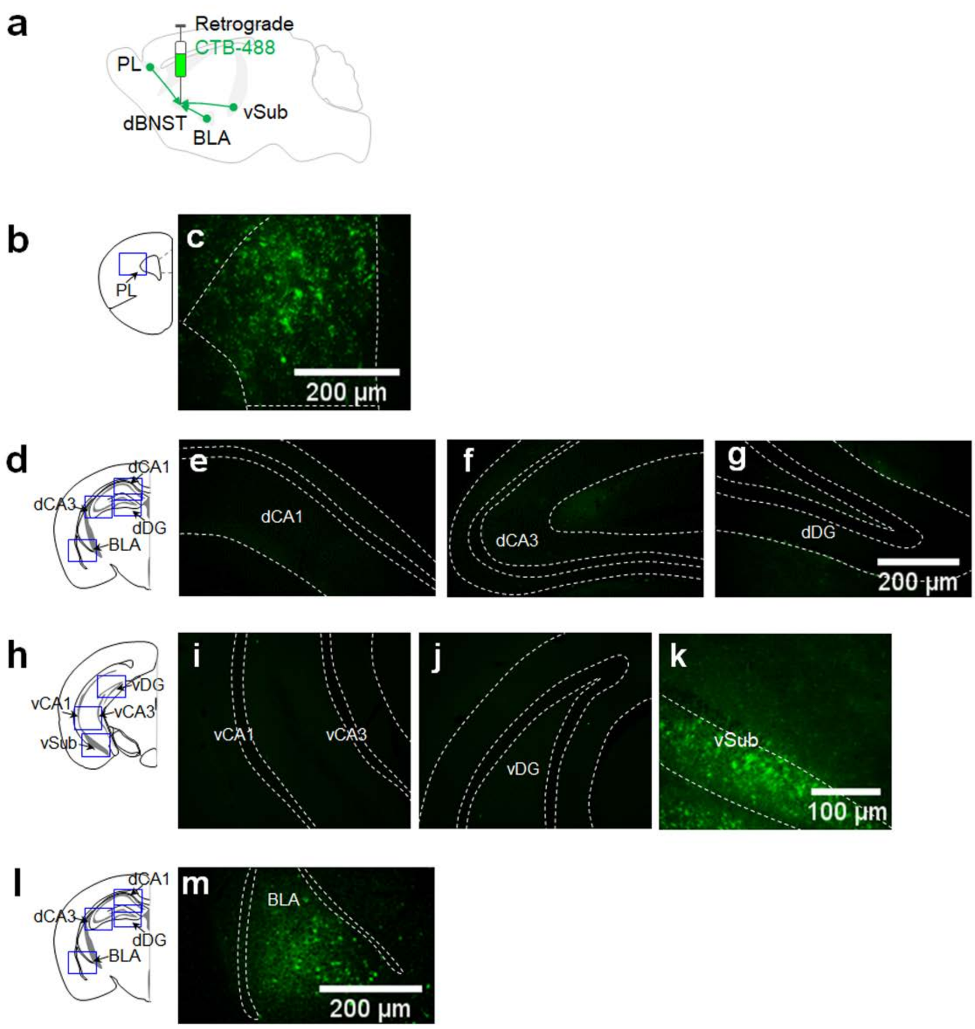

Supplementary Fig. 3. The dBNST receives neuronal inputs from the prelimbic cortex (PL), ventral subiculum (vSub), and basolateral amygdala (BLA).
a, Experimental design (**a**). CTB488 (a retrograde tracer) was injected into the dBNST and its expected tracking was depicted.

b–m, Diagrams illustrate the locations of photomicrographic images corresponding to the details in clonal sections (**b,d,h,i**). Representative photomicrographs showing CTB488 localization (green) in the prelimbic cortex (PL) (**c**), dorsal CA1 (**e**), dorsal CA3 (**f**), and dorsal dentate gyrus (**j**) regions of the dorsal hippocampus; the ventral CA1, ventral CA3 (**i**), ventral dentate gyrus (**j**), and ventral subiculum (vSub) regions of the ventral hippocampus; and the basolateral amygdala (BLA) (**m**). Parts of this figure (**a, k** panels) are identical to those shown in **Fig. 2a-e**.

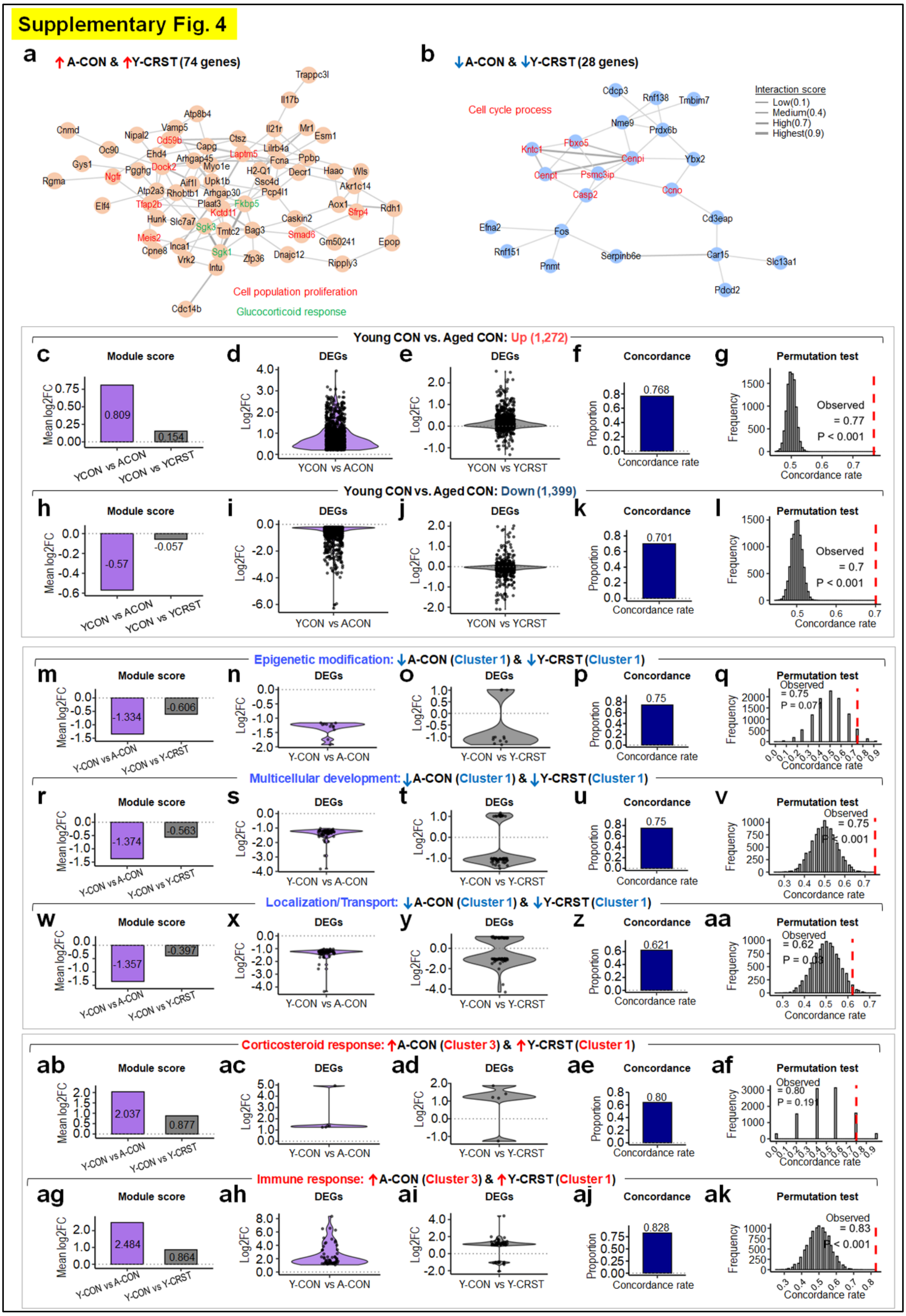

Supplementary Fig. 4. Aged mice exhibited vSub gene expression profiles similar to those induced by CRST in young mice.

a,b, Functional interaction networks of shared differentially expressed genes in the vSub. The networks of 74 upregulated genes (**a**) and 28 downregulated genes (**b**) (≧1.15-fold, p≦0.05) that were commonly altered in the vSub of both naïve aged mice and CRST-treated young mice, relative to young controls. Gene Ontology enrichment of these genes identified top BP terms, including those for corticosteroid response (Fkbp5, Sgk1, Sgk3), cell population proliferation (Ngfr, Smad6, ad Tfap2h), and cell cycle process (Fbxo5, Psmc3ip, Casp2).

c–**g**, Mean $\log_2$ fold change ($\log_2$FC) (**c**) and distribution (**d**,**e**) of the 1,272 genes upregulated in aged mice (relative to young controls), and the same genes in CRST-treated young mice (relative to young controls). Directional concordance of gene expression changes between the two groups (**f**), and its p-value (red-dashed line) (**g**) presented relative to a permutation-based null distribution.

h–**l**, Mean $\log_2$FC (**h**) and distribution (**i**,**j**) of the 1,399 genes downregulated in aged mice (relative to young controls), and the corresponding genes in CRST-treated young mice (relative to young controls). Directional concordance of gene expression changes between the two groups (**k**), and its p-value (**l**) against a permutation-based null distribution.

m–**aa**, Mean $\log_2$FC (**m,r,w**) and distribution (**n,o,s,t,x,y**) of GSVA-selected gene sets for epigenetic modification (**m**–**o**), multicellular development (**r**–**t**), and localization/transport (**w**–**y**) within the downregulated genes in both aged and CRST-treated young mice. Directional concordance (**p,u,z**) of gene expression change between two groups and its p-value (**q,v,aa**) shown relative to a permutation-based null distribution. Mean $\log_2$FC panels (**m,r,w**) are replicated in **Fig. 3j,k,l**.

ab–**ak**, Mean $\log_2$FC (**ab,ag**) and distribution (**ac,ad,ah,ai**) of GSVA-selected gene sets for corticosteroid response (**ab**–**ad**) and immune response (**ag**–**ai**) within the upregulated genes in both aged and CRST-treated young mice. Directional concordance (**ae,aj**) of gene expression changes between two groups and its p-value (**af,ak**) shown relative to a permutation-based null distribution. Mean $\log_2$FC panels (**ab,ag**) are replicated in **Fig. 3r,s,t**.

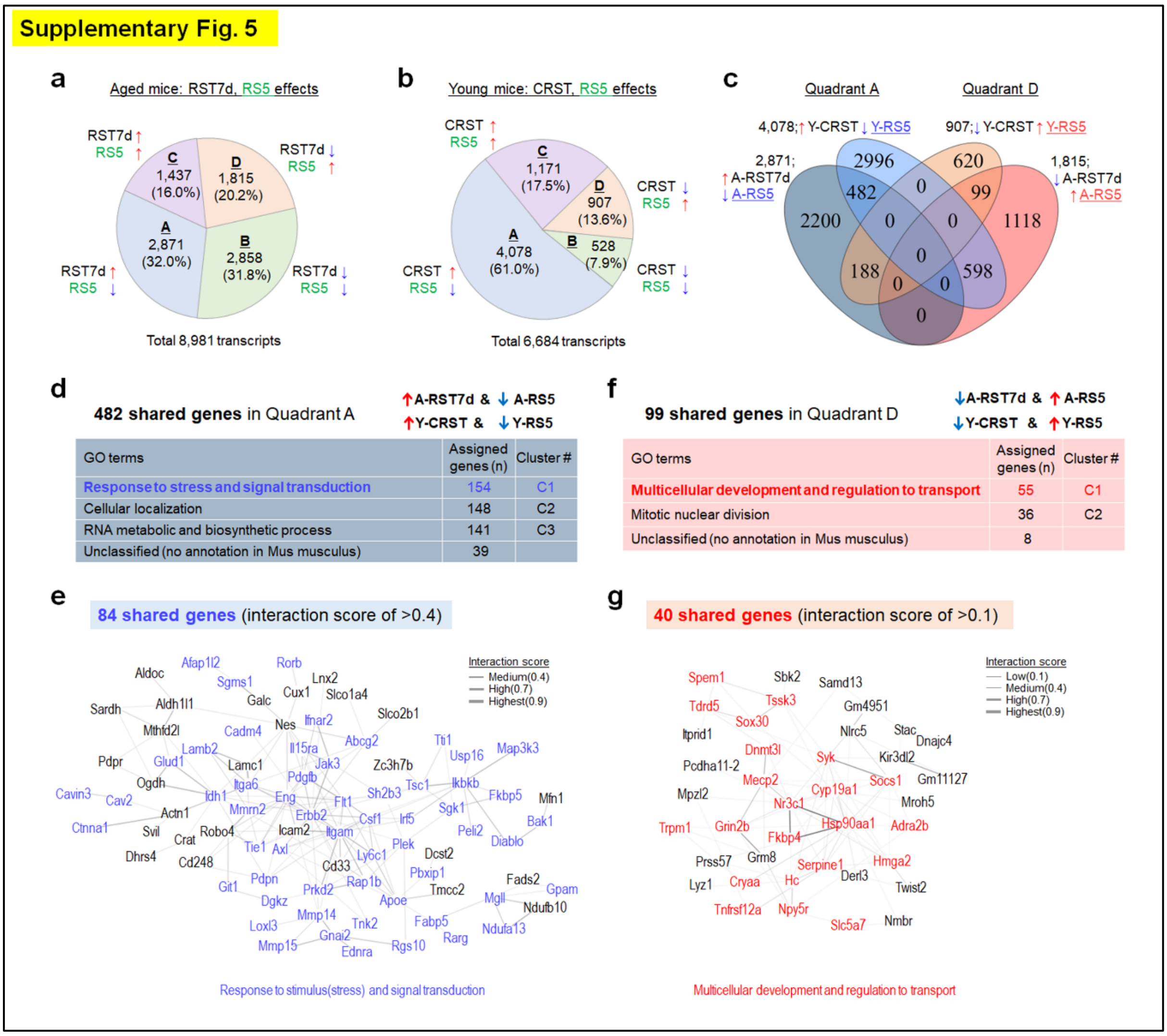

GO terms	Assigned genes (n)	Cluster #
Response to stress and signal transduction	154	C1
Cellular localization	148	C2
RNA metabolic and biosynthetic process	141	C3
Unclassified (no annotation in Mus musculus)	39	

GO terms	Assigned genes (n)	Cluster #
Multicellular development and regulation to transport	55	C1
Mitotic nuclear division	36	C2
Unclassified (no annotation in Mus musculus)	8	

Supplementary Fig. 5. Aged and young mice showed vastly divergent gene expression profiles for stress responses, with few commonalities.

a–c, Pie charts illustrating the number and percentage of genes within each of four quadrants. Chart (**a**) illustrates identified genes with expression changes after RST7d and subsequent RS5 in aged mice, while chart (**b**) displays those following CRST and subsequent RS5 in young mice (replicated from Fig. 3,4). A Venn diagram (**c**) showing the common and unique sets of genes altered under various conditions. This includes two comparisons (CON vs. RST7d; RST7d vs. RST7d+RS5) in aged mice, two comparisons (CON vs. CRST; CRST vs. CRST+RS5) in young mice, and all four comparisons between aged and young mouse groups.

d–g, The 482 shared genes in Quadrant A (**d**) and the 99 shared genes in Quadrant D (**f**) from both aged and young mouse groups, displayed with their functional clusters determined by k-means clustering. Functional interaction networks of genes shared between aged and young mouse groups: Functional interaction network (**e**) displays 84 shared genes (interaction score of >0.4) from Quadrant A, including 54 genes associated with stress response and signal transduction; and functional interaction network (**g**) shows 40 shared genes (interaction score of >0.1) from Quadrant D, including 22 genes involved in multicellular development and transport regulation.

Supplementary Fig. 6

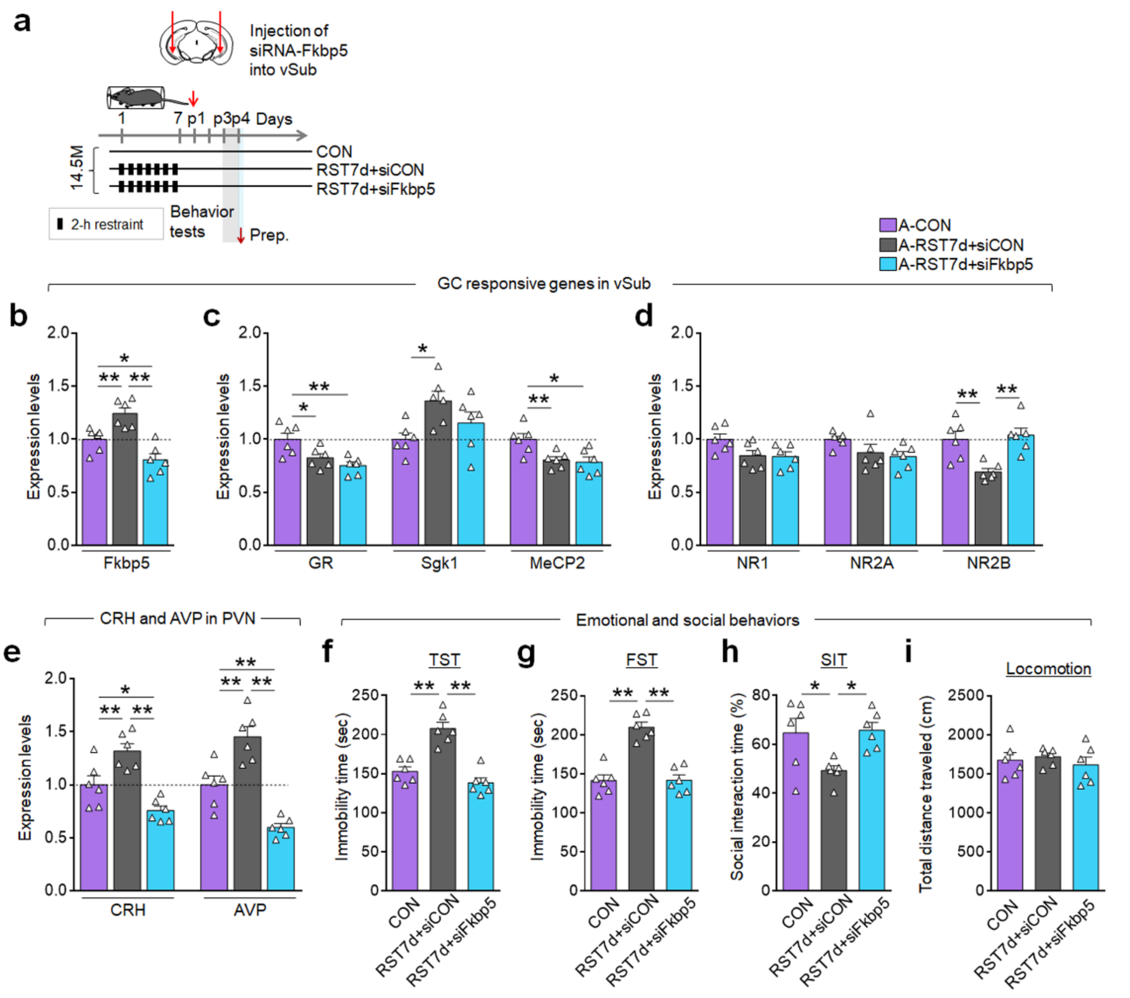

Supplementary Fig. 6. Fkbp5 knockdown within the vSub restored RST7d-induced physiological and behavioral deficits in aged mice.
a, Experimental design. Aged mice (14.5 M) were subjected to RST7d, followed by siRNA-Fkbp5 or siRNA-control injection into the vSub.

b–e, Transcript levels of Fkbp5 (**b**), GR, Sgk1, and Mecp2 (**c**); and NR1, NR2A, and NR2B (**d**) within the injected region (vSub), and CRH and AVP in the PVN (**e**) for the indicated groups.

f–i, The immobility time in the TST (**f**) and FST (**g**); Social interaction (% time) (**h**) and locomotor activity (**i**) in the SIT for the indicated groups.

Data are mean ± SEM. *,**, difference between indicated groups. *, $p < 0.05$; **, $p < 0.01$ (One-way ANOVA followed by Newman-Keuls post-hoc test). See Supplementary Data 4 for statistical details.

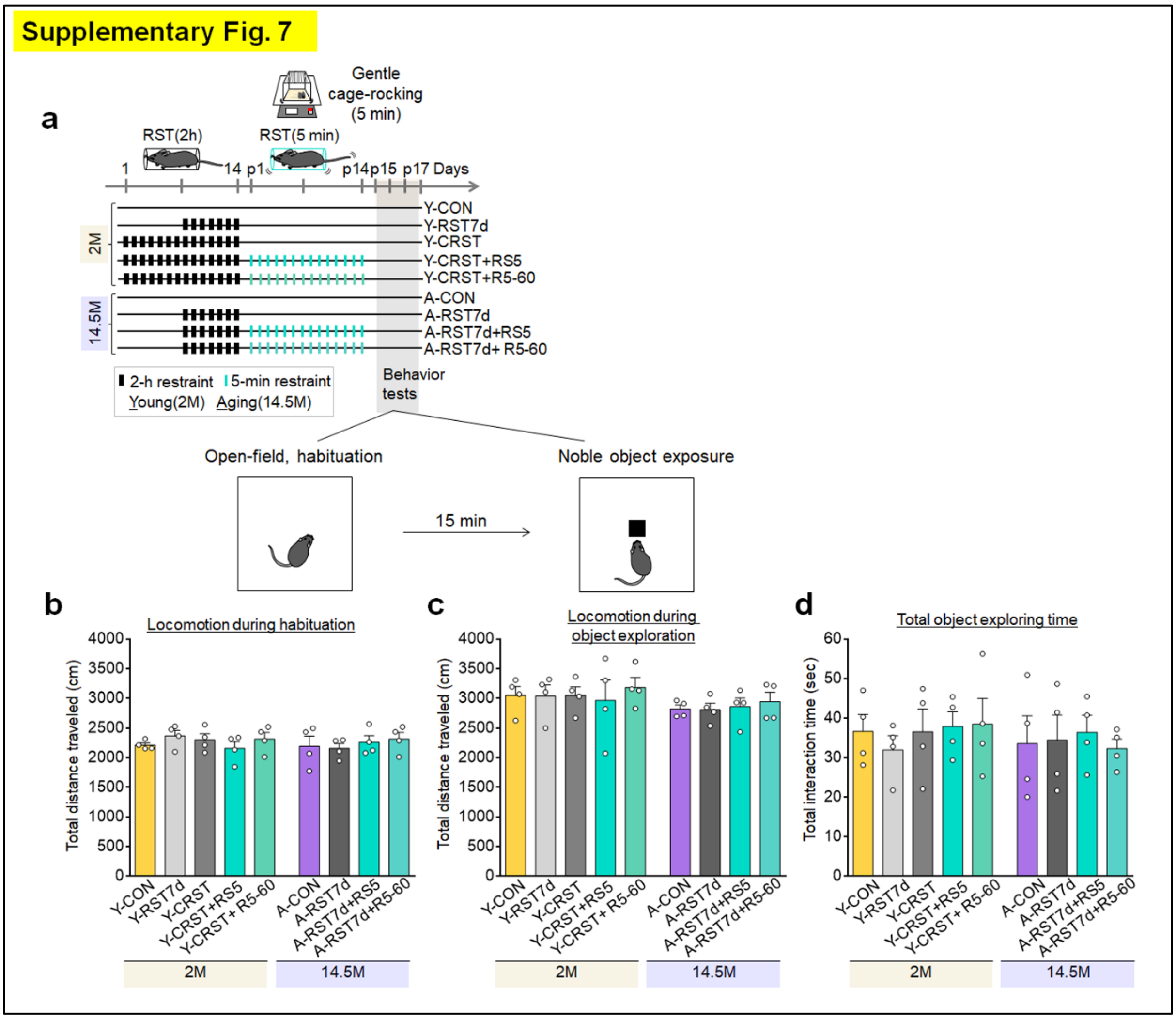

Supplementary Fig. 7. Normal motor and sensory-related behaviors in young and aged mice.

a, Experimental design. Young (2 M) and aged (14.5 M) mice were exposed to CRST or RST7d, respectively, as illustrated. Subsequently, CRST-treated young mice and RST7d-treated aged mice were subjected to repeated mild stress (5-min restraint (RS5) or 5-min gentle rocking at 60 rpm (R5-60)), as shown. Locomotor activity was recorded during the 10-min habituation phase in the open field, followed by a 15-minute interval. Subsequently, both locomotion and time spent exploring a novel object were measured during an additional 10-min test period.

b–d, Total distance traveled by mice in the open field during the 10-min habituation period (**b**); total distance traveled during the 10-min novel object exploration period (**c**); and total exploration time spent interacting with the object during the 10-min novel object exploration period (**d**) for the indicated young and aged mouse groups.

Data are mean ± SEM. (One-way ANOVA followed by Newman-Keuls post-hoc test). See Supplementary Data 4 for statistical details.

Supplementary Fig. 8

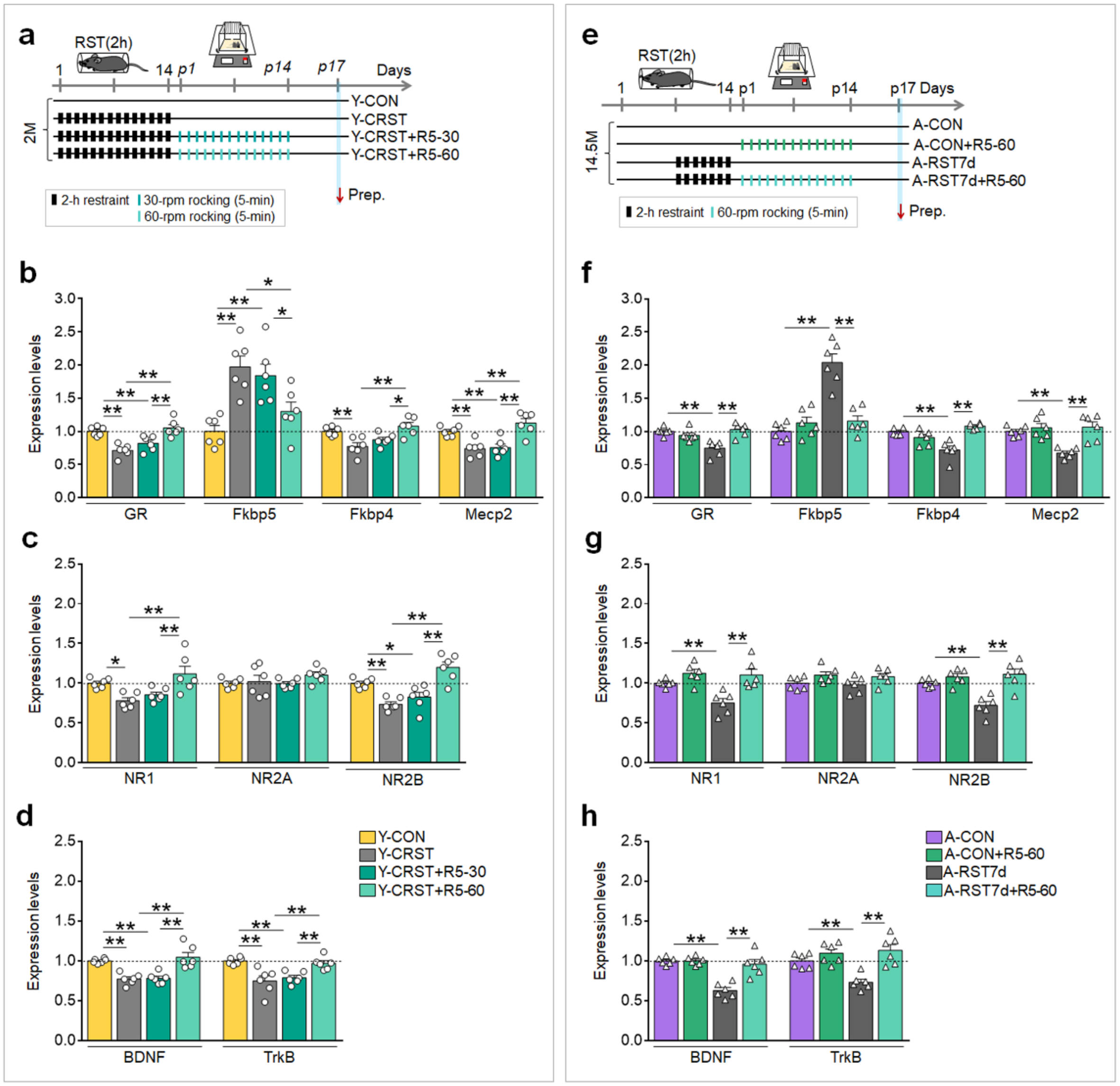

Supplementary Fig. 8. Repeated mild stress induced by gentle rocking reversed stress-induced gene expression changes in the vSub of young and aged mice. **a**, Experimental design. Young mice (2 M) subjected to 14 days of chronic restraint stress (Y-CRST; 2 hr day^{-1}) were treated with 5-min daily gentle rocking at 30 or 60 rpm for 14 days (Y-CRST+RS5-30 and Y-CRST+RS5-60, respectively) (*n* = 6 animals per group).

b–d, Transcript levels of GR, Fkbp5, Fkbp4, Mecp2 (**b**); NR1, NR2A, NR2B (**c**); and Bdnf and TrkB (**d**) in the vSub of the indicated groups.

.

e, Experimental design. Aged mice (14.5 M) were treated with a 14-day regimen of 5-min daily gentle rocking at 60 rpm (A-CON+RS5-60). A separate group of aged mice, subjected to 7 days of subchronic stress (A-RST7d, 2 hr day^{-1}), received 14 days of 5-min daily gentle rocking treatments at 60 rpm (A-RST7d+RS5-60).

f–h, Transcript levels of GR, Fkbp5, Fkbp4, Sgk1, and Mecp2 (**d**); NR1, NR2A, and NR2B (**e**); and Bdnf, and TrkB (**c**) in the vSub of the indicated groups.

Data are mean ± SEM. *.**, difference between indicated groups. *, $p < 0.05$; **, $p < 0.01$ (One-way ANOVA followed by Newman-Keuls post-hoc test). See Supplementary Data 4 for statistical details.

Supplementary Detailed Materials and Methods

Animals

Male 7-week-old C57BL/6 mice were purchased from Daehan BioLink (Eumsung, Chungbuk, Republic of Korea). Mice were housed in pairs in standard clear plastic cages containing wood chip bedding (TAPVEI, Paekna, Estonia). The environment was maintained under controlled conditions (23-24°C temperature, 50-60% humidity) with a 12-hour light/dark cycle, with lights on at 7:00 AM.). Food and water were provided to mice *ad libitum*. Mice were aged to the indicated points by housing 2-4 animals of the same gender per cage. All animal procedures were performed in accordance with Animal Ethics Committee of Ewha Womans University and were approved by the Institutional Animal Care and Use Committee (IACUC) at Ewha Womans University (IACUC19-015).

Chronic and subchronic restraint stress

Subchronic or chronic restraint was performed as described previously[7,18]. Mice were individually restrained in ventilated 50-ml conical tubes for 2 hours daily (10 AM - 12 PM) within their home cages, and this procedure was repeated for 7 days (subchronic restraint stress; RST7d) or 14 days (chronic restraint stress, CRST). After daily restraint session, mice were placed in their home cages with free access to food and water.

Repeated 5-min restraint or 5-min gentle rocking stress

Repeated 5-minute restraint was delivered as described previously[18]. Mice were individually placed in ventilated 50-ml conical tubes for 5 minutes for the indicated number of days. As a separate procedure of repeated mild stress, a method involving repeated 5-minute gentle cage-shaking was developed. In brief, repeated 5-minute sessions of gentle cage-shaking were administered by placing mice in their home cages on a rotary shaker (Hoefer Inc., San Francisco, CA, USA) at 30, 60, or 120 rpm for 5 minutes for the indicated days. Following each restraint or gentle cage-shaking session, mice were returned to their home cages with ad libitum access to food and water.

Behavioral assay

Behavioral tests were carried out as described previously[18,23,24]. Mice were randomly assigned to experimental groups. Mice were allowed to acclimate to the behavioral testing room for 20–30 minutes before the start of each test. The illumination level in the behavioral room was 20 lux for social behavior assessments and 250 lux for the Tail Suspension Test (TST) and Forced Swim Test (FST). Throughout all behavioral testing, a 65-dB white noise was used to mask background noise. For each behavioral test, subject mice were randomly ordered and positioned within the apparatus. Behavioral tests were recorded using either a video tracking system (SMART; Panlab S.I., Harvard Apparatus, USA) or a webcam recording system (HD Webcam #C210; Logitech, USA). All tests were conducted between 9:00 AM and 3:00 PM during the light cycle. The apparatus was thoroughly cleaned with 70% ethanol after each test.

Social interaction test and social recognition memory tests

The social interaction test (SIT) was performed as described previously[18,23]. The apparatus consisted of two symmetrical chambers (26 cm x 26 cm rectangular floor,

40 cm walls) connected by a middle intermediary passage (8 cm wide x 10 cm long x 26 cm high). A circular grid cage (12 cm diameter x 33 cm height) was placed in each symmetrical chamber.

For the social interaction test (SIT), a subject mouse was initially placed in the middle passage and allowed 5 minutes to explore and habituate to both chambers of the apparatus. Following a 2-minute return to its home cage, a social target mouse (A) was introduced into one of the grid cages within a chamber. Once the social target was stable, the subject mouse was again placed in the middle passage and allowed to freely explore for 10 minutes. During this exploration phase, one chamber contained the social target (A) in a grid cage, while the other chamber held an empty grid cage. The time spent in each chamber and the trajectory of the subject mouse were recorded. Sociability was defined as the relative amount of time the subject mouse spent exploring the chamber containing the social target compared to the chamber with the empty cage. This social interaction test (SIT) was followed by a series of social recognition memory tests (SMT) described below.

Five minutes after the sociability test, a novel social target (B) was introduced into the previously empty grid cage. Once this novel target was stable, the subject mouse from the SIT was placed back in the middle passage and allowed 10 minutes of free exploration. During this time, one chamber contained the now-familiar social target (A), while the other held the novel social target (B) in its grid cage. The time spent exploring the chamber with the novel social target (B) relative to the time spent exploring the chamber with the familiar social target (A) was recorded, and this constituted the 5-minute social recognition memory test. One hour later, the previous novel social target (B) was replaced with a second novel social target (C), and the 1-hour social recognition memory test was conducted similarly. The positions of the second novel social target (C) and the familiar mouse (A) were switched within the apparatus. The time spent exploring the chamber containing the second novel social target (C) and the time spent exploring the chamber containing the familiar mouse (A) were recorded. Twenty-four hours after the sociability test, the previous second novel social target (C) was replaced with a third novel social target (D), and the 24-hour social recognition memory test was performed similarly as above. The time spent exploring the chamber containing the third novel social target (D) and the time spent exploring the chamber containing the familiar mouse (A) were recorded. The position of the second novel social target and a familiar mouse were relocated with respect to the test room. The difference between the time spent exploring the chamber with a novel social target and the time spent exploring the chamber with a familiar mouse were regarded as social recognition memory, following the convention that a preference for the novel social target indicates recognition memory. Conversely, a preference for the familiar social target over the novel social target was defined as a social novelty preference.

Tail suspension test

The tail suspension test (TST) was carried out as described previously[18,24]. Mice were individually suspended 50 cm above the table surface by securing their tails to the top of a rectangular box with adhesive tape. The TST was performed for 6 min, and the total immobility time for each mouse was recorded. The immobility was defined as the complete cessation of body and limb movement.

Forced swim test
The forced swim test was performed as described previously[18,24]. The subject mice were placed in a transparent Plexiglass cylinder (15 cm in diameter x 27 cm in height) filled with water (a depth of 15 cm) at 24°C - 25°C for 6 min. During the 6 min test, the latency to the first immobility and the total immobility time during the final 5 min were measured. Immobility was defined as the state when an animal remained floating with motionless limbs.

Motor and sensory function tests
Motor and sensory functions of young and aged mice were assessed using a novel object exploration test in the open field. Each mouse was first allowed to explore an open field (45 × 45 cm) without an object for 10 minutes to habituate, during which total locomotor activity was recorded. After a 15-minute interval, the mice were reintroduced into the same open field containing a wooden block (3.5 × 3.5 × 7 cm) placed at the center for 10 minutes. During this session, total locomotion and the time spent exploring the object were measured. Object exploration was defined as the mouse approaching and making direct contact with the object using its nose or forepaws.

RNA seq analysis
RNA seq analysis was performed as described previously[18,25]. Brain tissue samples were homogenized with Trizol® reagent (#15596018; Invitrogen, Carlsbad, CA, USA) following the manufacturer's instructions. Total RNA was isolated using the RNeasy MinElute Cleanup kit (#74204; Qiagen, Hilden, Germany), and treated with DNase I (New England Biolabs, MA, USA) to ensure residual DNA. RNA sequencing and quality control processes were performed by Macrogen Inc. (Seoul, Republic of Korea). RNA quantification was carried out using the Quant-iT RiboGreen RNA Assay Kit (#R11490; Invitrogen, Carlsbad, CA, USA) with a Victor Nivo Multimode Microplate Reader (#HH350005500; PerkinElmer, Waltham, MA, USA). RNA quality was assessed using the 2100 Bioanalyzer (#G2939BA; Agilent, Santa Clara, CA, USA). RNA samples (400 ng) exhibiting 28S/18S rRNA ratios between 1.8 and 2.0 and an RNA integrity number (RIN) above 7.0 were used for sequencing library preparation. Total RNA was randomly fragmented, and cDNA was synthesized. Unique adaptors were ligated to both ends of the cDNA fragments, followed by PCR amplification to generate sufficient material for sequencing. Libraries were sequenced as 101 bp paired-end reads on a NovaSeq 6000 (Illumina Inc., CA, USA) using the Illumina TruSeq™ stranded mRNA kit (#20020594). Demultiplexed reads were evaluated for quality with FastQC, and after trimming with Trimmomatic 0.38 (Bolger et al., 2014), over 95% of the data reached a Q30 score. Trimmed reads were aligned using HISAT2 (version 2.1.0), and StringTie (version 2.1.3b) was used for transcript assembly.

RNA sequencing data processing
RNA-seq read counts per transcript were normalized using the trimmed mean of M-values (TMM) method, as implemented in the calcNormFactors function of the edgeR package (v4.0.16). Normalized log2-counts per million (logCPM) values were used for linear modeling with the lmFit function from the limma package (v3.58.1). Experimental group comparisons were modeled using a design matrix without an intercept, and contrasts were specified with the makeContrasts function. Moderated statistics were computed via empirical Bayes shrinkage using the eBayes function. This approach

yielded log2 fold changes and Benjamini–Hochberg adjusted p-values for each gene. A total of 45,777 genes were included in the differential expression analysis.

Gene set enrichment analysis

Gene set enrichment analysis was carried out as described previously[18,25]. Principal component analysis (PCA) was performed to assess sample-level transcriptomic variation among experimental groups in R (v4.3.2) using prcomp() with centering and unit variance scaling enabled. TMM-normalized log2-counts per million (logCPM) expression data, obtained from the edgeR package (v4.0.16), were used as input. The first two principal components were visualized using ggplot2 (v3.5.1), with the explained variance annotated on the axes.

Differentially expressed genes (DEGs) by aging or CRST were selected based on a fold change threshold of ≥ 1.15 (upregulation or downregulation) and a significance level of $p < 0.05$. Volcano plots of DEGs were generated for 45,777 signal values, with $\log_2$ fold-change for the x-axis and $-\log_{10}$ (p-value) for the y-axis, comparing the two experimental groups. Meanwhile, the differential gene expression for pairwise comparisons in both aged mice (CON vs. RST7d; RST7d vs. RST7d+RS5) and young mice (CON vs. CRST; CRST vs. CRST+RS5) was determined using a Rank-Rank Hypergeometric Overlap (RRHO) method (http://systems.crump.ucla.edu/rankrank/)[26,27] to process the signed log10-transformed p-values of RNA-seq signals. The top 50% of genes from each RRHO map quadrant were then selected for further analysis.

Selected genes were then grouped into functionally coherent clusters using k-means clustering. The optimal number of clusters (k) was selected based on three criteria: i) stable enrichment of relevant biological process (BP) terms within clusters, ii) minimal presence of irrelevant BP terms within a cluster, and iii) minimal overlap (high dissimilarity) of BP terms across different clusters. Gene Ontology (GO) enrichment analysis was performed on these clusters using the STRING database (v12.0; http://string-db.org) or gProfiler (https://biit.cs.ut.ee/gprofiler/gost). The hierarchy of GO biological process annotations was based on the Mouse Genome Database (http://www.informatics.jax.org). Additionally, Gene Set Variation Analysis (GSVA) was applied using the msigdbr (v7.5.1) and GSVA (v1.50.5) R packages. Finally, the results were visualized using the R packages, ggplot2 (v3.5.1), VennDiagram (v1.7.3), pheatmap (v1.0.12), and igraph (v2.1.1).

Differences in GSVA scores between comparison groups were assessed using one-way ANOVA, followed by post-hoc Tukey's Honest Significant Difference (HSD) test, with statistical significance set at $p < 0.05$. All analyses were performed using R (v4.3.2).

Directional differential gene expression between comparison groups was determined by comparing a $\log_2$ fold change ($\log_2$FC) threshold of ≥0.2. Module scores were calculated as the mean $\log_2$FC for DEGs within each cluster. Violin plots were generated to visualize the distribution of gene-level changes.

Directional concordance between aging- and stress-related DEGs was quantified as

the proportion of genes showing the same direction of change. Statistical significance was determined by permutation testing (10,000 iterations), with random reassignment of up/down labels to generate a null distribution. The empirical p-value was calculated as the fraction of permutations, yielding a concordance rate equal to or exceeding the observed rate. All analyses were performed using R (v4.2.2) with the ggplot2 (v3.4.2) package for visualization and base R functions for statistical computations.

Gene knockdown and gene overexpression experiments in HT22 cells

Mouse HT22 cells were cultured as described previously[18]. Briefly, HT22 cells were maintained in Dulbecco's Modified Eagle Medium (DMEM) containing 4500 mg/L D-glucose, L-glutamine and 100 mg/L sodium pyruvate (#LM001-05; Welgene) supplemented with 10% heat-inactivated fetal bovine serum (FBS) (#F2442, Sigma-Aldrich) and antibiotics (penicillin and streptomycin) (#LS202–02, Welgene) at 37°C in a humidified incubator with 95% air and 5% CO_2. HT22 cell were seeded in a 6-well plate at a density of 1.0 x 10^5 cells per well. Following growth to 70–80% confluency, cells were treated for 24 hours with corticosterone (400 ng per ml; #C2505, Sigma-Aldrich) in DMEM containing 1% FBS, prior to harvesting.

Transfection of siRNA-target gene and plasmid-Mecp2 DNA into HT22 cells was performed as described previously[7,28]. Briefly, HT22 cell were seeded in a 6-well plate in DMEM containing 1% FBS. Lipofectamine 2000 (#11668019, Invitrogen) (9 μl) and 20 μM siRNA (3 μl) or 20 μM plasmid-Mecp2 DNA (3 μl) were separately diluted in 150 μl of Opti-MEM® Medium (31985070, Gibco, Thermo Fisher Scientific, Paisley, Scotland, UK). The final concentration of siRNA was 30 nM or of plasmid DNA was 0.125 μg.

The siRNA-control (#SN-1012), siRNA-GR (#14815, NM_008173.3), siRNA-Fkbp5 (#14229, NM_010220.4), siRNA-Sgk1 (#20393, NM_001161845.2), siRNA-Mecp2 (#17257, NM_010788.4), and siRNA-Hdac2 (#15182, NM_008229.2) were purchased from Bioneer Co. (Daejeon, Korea). The pCNS-D2-Mecp2 plasmid (KU021747) was obtained from Korea Human Gene Bank, Medical Genomics Research center, KRIBB, Republic of Korea.

Chromatin immunoprecipitation-qPCR analyses

Chromatin immunoprecipitation (ChIP)-qPCR assay was performed as described previously[7] using a ChIP-IT Express Kit (#102026, Active Motif). In brief, HT22 cells grown to 70–80% confluence in 100-mm dishes were treated with drugs or reagents. After washing with 1X PBS, cells were treated with 1% formaldehyde in 1X PBS for 10 min to cross-link proteins and DNA. The cross-linking reaction was quenched by adding 3 ml of 0.125 M glycine. Cells were homogenized, and then resuspended in lysis buffer containing a proteinase cocktail (0.5X final) and PMSF (0.5 mM final) and processed according to the ChIP-IT Express Kit protocol. Subsequently, the fixed chromatin pellet was resuspended in 350 μl of shearing buffer containing proteinase inhibitors and PMSF. To fragment the chromatin into 200-800 bp, sonication was performed on ice using an Epishear probe sonicator (#53052, Active Motif, Carlsbad, CA, USA) with 20 pulses of 20 seconds each, separated by 50-second intervals at 35% power. Finally, the sheared chromatin samples were centrifuged at 22,000 g for 10 minutes, and the resulting supernatant was used for immunoprecipitation.

The DNA concentration of the sonicated sheared chromatin was quantified using a NanoDrop spectrophotometer (#ND-2000, Thermo Fisher Scientific). The DNA concentration of the sheared chromatin was determined using a NanoDrop (#ND-2000, Thermo Fisher Scientific), and the expected fragment size range (200-800 bp) was confirmed via agarose gel electrophoresis. For each immunoprecipitation, 10 µg of sheared chromatin was incubated overnight at 4 °C with 2 µg of primary antibody (Mecp2, #3456, Cell Signaling Technology), 20 µl of Protein G magnetic beads, 10 µl of 10X ChIP buffer 1 (ChIP Assay Kit, #102026, Active Motif), and 1 µl of proteinase inhibitor cocktail in a total volume of 100 µl.

The immunoprecipitated products were washed with ChIP buffer I and subsequently with ChIP buffer II using a magnetic rack (#102026, Active Motif). After washing, the bound DNA complexes were eluted with 50 µl of elution buffer. To release the DNA, 50 µl of reverse cross-linking buffer was added, and the supernatant containing the eluted chromatin was incubated at 95 °C for 15 minutes. In parallel, a 10 µl aliquot of the saved sheared input DNA was also subjected to reverse cross-linking under the same conditions. Both ChIP and input DNA samples were then treated with 1 µg of proteinase K for 1 hour at 37 °C, and the digestion was terminated with 2 µl of proteinase K stop solution. These resulting DNA samples were used for real-time PCR. The primer sets were Fkbp5 P1-F (5′-ACATCGCATTTTCCCAGTCT-3′), Fkbp5 P1-R (5′-CTTCACGAGCTGCAGAGGTC-3′), Fkbp5 P2-F (5′-GCCATCTCGAAGCTGACCTA-3′), Fkbp5 P2-R (5′-AGGTTTCTGCCTCTCCAACA-3′), and GAPDH-F (5′-AGAAGGTGGTGAAGCAGGCAT-3′), and GAPDH-R (5′-CGAAGGTGGAAGAGTGGGAGTTG-3′).

Immunofluorescence staining

Immunofluorescence staining of brain sections or HT22 cells was carried out as described previously[7,18]. In brief, mice were anesthetized with a mixture of ketamine hydrochloride (50 mg ml^{-1}) and xylazine hydrochloride (23.3 mg ml^{-1}) (3.5: 1) at a dose of 2.5 µl g^{-1} body weight, and then transcardially perfused with 4% paraformaldehyde. The brains were then carefully isolated and post-fixed in 4% paraformaldehyde overnight at 4°C. Coronal sections (40 µm) were cut using a vibratome (VT1000S, Leica). The sections were then blocked for 1 hour in 4% BSA (#22070008, BioWorld) in 1X PBST (#1610407, Bio-Rad), followed by overnight incubation at 4°C with primary antibody in 4% BSA. After washing in 1X PBST, the sections were incubated with fluorescence-tagged secondary antibodies diluted 1:500 in 1X PBST. Subsequently, the stained sections were mounted with DAPI-containing antifade medium (Vector Laboratories, #H-1800). Immunofluorescence images were visualized using an Olympus BX 51 microscope with a DP71 camera, and were analyzed using MetaMorph Microscopy Automation & Image Analysis software (Molecular Devices, Sunnyvale, CA, USA).

The primary antibodies included anti-c-Fos (#sc-271243, 1:300, Santa Cruz Biotechnology), anti-GR (#CST-12041S, 1:500, Cell Signaling Technology), anti-Fkpb5 (#GTX84491, 1:50, Genetex), and anti-Mecp2 (#GTX22828, 1:200, Genetex; 3456, 1:500, Cell Signaling Technology). The secondary antibodies included anti-rabbit IgG DyLight488 (#DI-1488; 1:500, Vector Laboratories), anti-rabbit IgG DyLight594 (#DI-1094; 1:500, Vector Laboratories), anti-mouse IgG DyLight488 (#DI-2488; 1:500, Vector Laboratories), and anti-mouse IgG DyLight594 (#DI-2594; 1:500,

Vector Laboratories).

Real-time PCR analysis

Real-time PCR was carried out as described previously[7,18]. Total RNA was purified from brain tissue or HT22 cells using TRIzol reagent (#15596018, Invitrogen Life Technologies, Carlsbad) and treated with DNase I (#M610A, Promega, MO, USA). Total RNA (1 μg) was reverse-transcribed in a 20 μl reaction using a reverse transcription system (#A3500, Promega, Madison, WI, USA). Real-time PCR was performed in a 20 μl volume containing 10 μl of 2X iQ™ SYBR Green Supermix (#1708882, Bio-Rad Laboratories, Hercules, CA, USA), 1 μl each of 5 pmol/μl forward and reverse primers, and 4 μl of cDNA (a 1:8 dilution of the reverse transcription product). A CFX 96 Real-Time PCR System Detector (Bio-Rad Laboratories) was used for amplification, and the Bio-Rad CFX Manager 3.1 software was used for data analysis.

The following primer sets were used: CRH, 5'-CACCTACCAAGGGAGGAGAA-3' and 5'-GCAGGCAGGACGACAGAG; AVP, 5'-GCTCAACACTACGCTCTC-3' and 5'-CTTGGGCAGTTCTGGAAG; Nr3c1(GR), 5'-GGAATAGGTGCCAAGGGTCT-3' and 5'-CACGTCAGCACCCCATAATG; Fkbp5, 5'-AGCCGTACTTCACTCTCCGT-3' and 5'-AAGCCCCATCCTTTATGGCG; Fkbp4, 5'-GGCTCCGAAGTTGATAGCAG-3' and 5'-GCTTGCCTCAATCTCTGGTC; Hsp90aa1, 5'-GCCTGTTTGGAAAGGTGGTA-3' and 5'-TCCACCTTGAAAGGCAAATC; Sgk1, 5'-CGGAAAGGGCAGTTTTGGAA-3' and 5'-AGCGGTCTGGAATGAGAAGT; Mkp1, 5'-AGTGGAGATGAAAGGCACGA-3' and 5'-GACTTGGGTAGAGCTCCACA; Hdac2, 5'-GGGACAGGCTTGGTTGTTTC-3' and 5'-GAGCATCAGCAATGGCAAGT; Sirt1, 5'-GATCCTTCAGTGTCATGGTTC-3' and 5'-ATGGCAAGTGGCTCATCA; Mecp2, 5'-ACAGCGGCGCTCCATTATC-3' and 5'-CCCAGTTACCGTGAAGTCAAAA; NR1, 5'-TTCACAGAAGTGCGATCTGG-3' and 5'-GGACAGGGACACATTTTGCT; NR2A, 5'-CTCCTTGGTCACCACCATCT-3' and 5'-TCAGCTGGACCTGTGTCTTG; NR2B, 5'-GTGAGAGCTCCTTTGCCAAC-3' and 5'-AGGGTAGAGCGACTTGCTGA; BDNF, 5'-TGGCTGACACTTTTGAGCAC-3' and 5'-GTTTGCGGCATCCAGGTAAT; TrkB, 5'-AAGGACTTTCATCGGGAAGCTG-3' and 5'-TCGCCCTCCACACAGACAC; and L32, 5'-GCTGCCATCTGTTTTACGG-3' and 5'-TGACTGGTGCCTGATGAACT.

Serum corticosterone measurement

Serum corticosterone levels were quantified as described previously[18] using an ELISA kit (ADI-901-097; Enzo Life Science, Farmingdale, NY, USA). Mice were anesthetized using 2.5% avertin (2,2,2-tribromoethanol; #T48402, Sigma-Aldrich) at a dose of 20 $\mu g\ g^{-1}$ body weight. Blood samples were collected from the abdominal aorta between 8 AM and 12 PM. Serum was obtained by centrifugation (3,000 g, 4°C, 15 min) and stored at -80°C. For the ELISA, each serum sample was treated with a steroid displacement reagent to release bound corticosterone and then diluted with assay buffer of the kit. Samples and standards were loaded onto an anti-sheep IgG-coated plate, and alkaline phosphatase and a sheep polyclonal anti-corticosterone antibody were added. Following antibody incubation, the plate was washed three times with washing buffer, and 200 μl of the kit's p-nitrophenyl phosphate substrate solution was added to each well. After a 1-hour incubation at room temperature, the reaction was stopped. Absorbance was read at 405 nm using a SpectraMax® M5 spectrofluorometer (Molecular Devices, Sunnyvale, CA, USA) and SoftMax Pro

software (version 5.4). Corticosterone concentrations were then analyzed using the Enzo Life Sci. ELISA data analysis tool (http://www.myassays.com).

Neural circuit mapping using retro-grade tracers

Neural circuit mapping with retro-grade tracers was carried out as described previously[18 8]. Mice were anesthetized with a mixture of ketamine and xylazine, as described above. To visualize neuronal afferents to the dBNST, 0.3 μl of the retrograde tracer Alexa Fluor 488-conjugated cholera toxin subunit B (CTB) (0.1%; #C34755, Molecular Probes, Eugene, OR, USA) was stereotaxically injected into the dBNST (AP, +0.25 mm; ML, -1.15 mm; DV, -4.2 mm). One week after stereotaxic injection, mice were transcardially perfused with 4% paraformaldehyde, and their brains were isolated and coronally sectioned at 40 μm using a vibratome (Leica VT 1000S). Fluorescence images were obtained and analyzed using an Olympus BX 51 microscope equipped with an X-cite 120 fluorescence illuminator (EXFO Life Science & Industrial Division, Ontario, Canada), a DP71 camera (Olympus), and MetaMorph Microscopy Automation & Image Analysis software (Molecular Devices).

Chemogenetic manipulation of specific neuronal circuits

Chemogenetic modulation of neurons was carried out using a DREADDs system as described previously[18,27]. Mice were anesthetized with a mixture of ketamine and xylazine. To chemogenetically activate vSub neurons, AAV8-CaMKIIα-hM3D(Gq)-mCherry (3×10^{10} viral particles ml^{-1}) was bilaterally injected into the vSub (AP, -3.5 mm; ML, ±2.75 mm; DV, -4.5 mm) using a stereotaxic injection system (Vernier Stereotaxic Instrument, Leica Biosystems, Wetzlar, Germany) and a Hamilton syringe with a 30-gauge needle. Following a 7-day recovery period, mice underwent chronic restraint stress test (CRST) in young mice or subchronic restraint stress (RST7d) in aged mice received an intraperitoneal injection of either vehicle (0.9% saline) or clozapine-N-oxide (CNO) (3 mg kg^{-1}; Tocris Bioscience, #6329) 30 minutes prior to a 5-minute restraint session (120 μl injection volume).

To chemogenetically inhibit vSub neurons projecting to the dBNST, a Cre-dependent inhibitory DREADD virus AAV8-hSyn-DIO-hM4D(Gi)-mCherry (3×10^{10} viral particles ml^{-1}), was bilaterally injected into the vSub (AP, -3.5 mm; ML, ±2.75 mm; DV, -4.5 mm). Simultaneously, a retrograde Cre-expressing virus, AAV8-EF1α-mCherry-IRES-WGA-Cre (1.5×10^{10} viral particles ml^{-1}), was injected into the dBNST (AP, +0.25 mm; ML, ±1.15 mm; DV, -4.2 mm). To induce inhibition of these vSub-to-dBNST projecting neurons, mice received an intraperitoneal injection of CNO (3 mg kg^{-1}) 30 minutes prior to a 5-minute restraint. The injection volume and rate for the vSub were 0.8 μl at 0.4 μl min^{-1}, and for the dBNST were 0.3 μl at 0.15 μl min^{-1}. Following each injection, the needle remained in the injection site for 5 minutes to facilitate adequate viral delivery and was then slowly retracted over a 5-minute period.

The AAV8-CaMKIIα-hM3D(Gq)-mCherry and AAV8-hSyn-DIO-hM4D(Gi)-mCherry viral vectors were obtained from Dr. Bryan Roth (Addgene: plasmids #50476 and #44362, respectively). The AAV8-EF1α-mCherry-IRES-WGA-Cre was purchased from UNC Vector Core (University of North Carolina, NC, USA). Upon arrival, all viral vectors were aliquoted and stored at -80°C until use. Each vector was diluted in 1x PBS (13.7 mM NaCl, 0.27 mM KCl, 0.8 mM Na_2HPO_4, 0.2 mM KH_2PO_4, pH 7.4).

siRNA-mediated gene knockdown in the vSub

Stereotaxic injection of siRNA was performed as described previously[7,18]. Briefly, mice were anesthetized with a mixture of ketamine and xylazine. Diluted siRNA (50 ng ml^{-1}) was mixed with Neurofect transfection reagent (#T800075, Genlantis, San Diego, CA, USA) at a 1:2.5 ratio and with sucrose (50% final concentration at 0.5 volumes), followed by a 20-minute incubation. A total of 0.5 μl containing 5.94 ng of siRNA-Fkbp5, or siRNA-control was bilaterally injected into the ventral subiculum (vSub; AP, -3.5 mm; ML, ±2.75 mm; DV, -4.5 mm) at a rate of 0.4 μl/min using a stereotaxic injection system (Vernier Stereotaxic Instrument) with a 30-gauge needle. Forty-eight to seventy-two hours following the siRNA injection, behavioral testing was performed and brain tissues were collected for knockdown or gene expression analyses. The siRNA-control (#SN-1012), and siRNA-Fkbp5 (#14229, NM_010220.4) were purchased from Bioneer Co. (Daejeon, Korea).

Statistical analysis

Statistical analyses were performed using GraphPad Prism 6 software (GraphPad Software, Inc., CA, USA). Two-sample comparisons were performed using two-sided Student's t-test, and multiple comparisons were analyzed with one-way ANOVA followed by the Newman-Keuls post-hoc test. All data are presented as mean ± SEM, and statistical differences were accepted at $p < 0.05$ unless otherwise indicated. The statistical details of the results of all main figures and Supplementary figures are provided in Supplementary Data 4.
